# Multiorgan and gut microbial alterations in ovariectomized mice: a multiomics analysis

**DOI:** 10.3389/fimmu.2026.1847257

**Published:** 2026-09-09

**Authors:** Chongwen Ma, Shuanhu Lei, Guangzhi Zhang, Xuchang Hu, Xiaoming Qiu, Qi Wang, Xuewen Kang

**Affiliations:** 1 Department of Orthopedics, Lanzhou University Second Hospital, Lanzhou, China; 2 The Second Clinical Medical College, Lanzhou University, Lanzhou, China; 3 Key Laboratory of Orthopedics Disease of Gansu Province, Lanzhou University Second Hospital, Lanzhou, China; 4 Gansu Provincial Hospital of Traditional Chinese Medicine, The First Affiliated Hospital of Gansu University of Chinese Medicine, Gansu University of Chinese Medicine, Lanzhou, China; 5 Cuiying Biomedical Research Center, Lanzhou University Second Hospital, Lanzhou, China

**Keywords:** estrogen deficiency, gut microbiota, gut-liver axis, multi-omics, ovariectomy, postmenopausal metabolic dysfunction

## Abstract

**Introduction:**

Postmenopausal metabolic dysfunction is increasingly recognized as a multisystem disorder associated with estrogen deficiency, yet how gut microbial, metabolic, and tissue-level alterations co-occur across organs remains incompletely characterized.

**Methods:**

Here, we used an ovariectomy (OVX) mouse model and an integrated multiomics strategy to characterize systemic alterations in gut microbiota, metabolites in colonic contents and circulation, and tissue-level molecular profiles across the colon, liver, skeletal muscle, and bone.

**Results:**

OVX mice showed higher body weights at multiple postoperative time points, lower serum estradiol concentrations, differences in selected inflammatory and bone-turnover markers, representative histological differences across multiple tissues, and OVX–Sham differences in femoral microarchitecture. Shotgun metagenomic profiling showed significant differences in gut microbial composition, with lower evenness-sensitive diversity and differences in dominant taxa. Metabolomic profiling of colonic contents demonstrated global differences in the luminal metabolic profile, including lower relative abundances of major short-chain fatty acids, differences in bile acid composition, and prominent tryptophan-related features. At the host interface, colonic transcriptomic analysis identified annotations related to epithelial membrane polarity, vesicle trafficking, endoplasmic reticulum protein processing, and bile acid- and energy-sensing pathways. Among the 19 differential circulating bile acids and short-chain fatty acids, most were lower in OVX mice. Feature-level analyses identified multiple hepatic metabolite and bile acid differences, whereas the hepatic transcriptome did not show significant global separation; differential-expression and gene-set enrichment analyses nonetheless identified selected differences related to lipid metabolism, energy metabolism, and molecular transport. Distal tissues also displayed molecular differences, including a significant global transcriptomic difference in skeletal muscle and a significant global metabolomic difference in bone; differential bone metabolites were annotated to energy-, amino-acid-, lipid-, and cyclic guanosine monophosphate–protein kinase G (cGMP–PKG)-related pathways.

**Discussion:**

Collectively, these findings define a gut-associated, multiorgan pattern of OVX-related remodeling characterized by concurrent microbial, metabolite, and tissue-level differences. This descriptive, associative, and hypothesis-generating dataset provides a reference for future studies testing the relevance of these OVX-associated patterns to menopause-associated metabolic dysfunction.

## 1 Introduction

Postmenopausal metabolic dysfunction comprises a group of metabolic abnormalities that emerge or worsen following ovarian functional decline and reduced estrogen levels (1). It is characterized primarily by central adiposity, insulin resistance, dyslipidemia, and elevated blood pressure, and it develops progressively during the menopausal transition (2). This condition poses a major threat to the health of postmenopausal women because it not only disrupts glucose and lipid homeostasis but also is closely associated with increased cardiovascular risk and functional alterations in multiple tissues, including the liver and skeletal muscle (3). Although estrogen deficiency is recognized as a central contributor, additional mechanisms may include altered intestinal permeability and host–microbiota interactions involving estrogen recirculation (4, 5). However, the concurrent microbial and molecular patterns across the gut and metabolically relevant tissues in the context of ovarian hormone loss remain incompletely characterized.

The gut microbiota is an important component of metabolic homeostasis and immune balance (6). It is involved in nutrient metabolism, mucosal immune regulation, and the turnover of steroid-related compounds (7). Recent advances in multiomics approaches, including microbiome profiling, transcriptomics, and metabolomics, have provided new opportunities to examine molecular patterns associated with postmenopausal metabolic dysfunction (8). Increasing evidence indicates that menopausal status is associated with differences in gut microbial composition and estrogen-related microbial functions, collectively referred to as the estrobolome, and that these differences are associated with adverse cardiometabolic phenotypes (9). In muscle-related studies, Huang et al. reported that rice bran supplementation in high-fat diet-fed ovariectomized (OVX) mice improved gut microbial dysbiosis and attenuated muscle atrophy (10). In liver-related studies, Liu et al. showed that estrogen deficiency was associated with gut microbial dysbiosis, reduced butyrate availability, and more severe fatty liver phenotypes (11). In studies of hormonal regulation, Chen et al. found that *Lactobacillus gasseri* CCFM1255 supplementation in OVX rats increased circulating estradiol levels and reshaped both the gut microbiota and the serum metabolome (12). In bone-related studies, Yu et al. demonstrated that microbiota-driven migration of intestinal tumor necrosis factor (TNF)-positive T cells and T helper 17 (Th17) cells into the bone marrow contributed to OVX-induced trabecular bone loss (13). Collectively, these findings support the relevance of gut microbial alterations to OVX-associated metabolic dysfunction across muscle, liver, hormonal regulation, and bone metabolism. However, most studies focus on a single organ or pathway, and coordinated profiling of gut microbial composition, metabolites in colonic contents and circulation, and molecular alterations across multiple organs within a common experimental framework remains limited.

Taken together, previous studies have identified selected features of postmenopausal metabolic dysfunction at the levels of gut microbial composition, microbial metabolism, tissue transcriptomics, and host metabolomics. Although integrated microbiome–metabolome studies have characterized microbial–metabolic relationships in clinical and experimental gastrointestinal settings (14, 15), systematic investigation of the relationship between gut dysbiosis and multitissue molecular alterations within the same OVX model remains limited. In this study, we used OVX mice to comprehensively characterize differences in the gut microbiota, metabolites in colonic contents, serum metabolites, the transcriptomes of the colon, liver, and skeletal muscle, and the metabolomes of the liver and bone. Through this multilayered design, we aimed to provide a descriptive and associative multiorgan overview of co-occurring molecular patterns under OVX-induced estrogen deficiency.

## 2 Materials and methods

### 2.1 Ethics statement

The animal experiments were conducted in accordance with the guidelines prescribed by the Chinese Association for Laboratory Animal Sciences and were approved by the Ethics Committee of Lanzhou University Second Hospital (Approval No. D2026-140).

### 2.2 Animals and experimental design

This study aimed to investigate the multiomics features of ovariectomy-induced estrogen deficiency, including gut microbiota, gene expression in the colon, liver, and skeletal muscle, metabolites in colonic contents and circulation, and liver and bone metabolomes in OVX mice and sham-operated controls. The individual mouse was the experimental unit. A total of 12 mice were included in each group. Female C57BL/6J mice were acclimatized in a temperature-, humidity-, and light-controlled specific pathogen-free animal facility for 1 week. After acclimation, mice at 7 weeks of age were randomly divided into two groups. The OVX group underwent bilateral ovariectomy, whereas the Sham group underwent sham surgery. After surgery, the mice were maintained for a further 9 weeks. Body weight was recorded weekly during postoperative weeks 1–5 and every 2 days during postoperative weeks 6–9.

### 2.3 Sample collection

At the end of the experiment, the mice were fasted for 12 h and then subjected to anesthesia, euthanasia, and biological sample collection. Samples were processed without group identifiers during downstream multiomics and biochemical analyses. Blood, small intestine, colon, pancreas, liver, inguinal white adipose tissue (iWAT), interscapular brown adipose tissue (BAT), skeletal muscle, bilateral femurs, and colonic contents were collected for downstream analyses. Colonic contents were collected for shotgun metagenomic sequencing and untargeted metabolomic analysis, including bile acid (BA) and short-chain fatty acid (SCFA) analyses. Blood samples were collected for serum preparation and subsequent untargeted metabolomics, BA and SCFA quantification, and enzyme-linked immunosorbent assay (ELISA) measurements. Colon, liver, and skeletal muscle tissues were collected for transcriptomic analysis. Liver tissue was additionally collected for untargeted metabolomics and BA analysis, whereas bilateral femurs were collected for bone metabolomic analysis. Small intestine, colon, pancreas, liver, distal femur, skeletal muscle, iWAT, and BAT were collected for histological examination. Collected samples were immediately stored at −80 °C or in liquid nitrogen and, after all samples had been collected, were transported on dry ice for downstream analyses.

### 2.4 Outcome measures

Body weight, serum hormone levels, inflammatory and metabolic indicators, bone turnover markers, histological alterations in multiple tissues, gut microbiota composition, colonic-content metabolomic profiles, circulating BA and SCFA profiles, and transcriptomic and metabolomic changes in the colon, liver, skeletal muscle, and bone were used as the main outcome measures.

### 2.5 ELISA analysis, histological examination, and micro-CT analysis

Mouse ELISA kits were used to detect the levels of estradiol (E2), monocyte chemoattractant protein-1 (MCP-1), tumor necrosis factor-α (TNF-α), interleukin-6 (IL-6), interleukin-1β (IL-1β), interleukin-10 (IL-10), adiponectin (ADPN), procollagen type I N-terminal propeptide (PINP), osteocalcin (OCN), C-terminal telopeptide of type I collagen (CTX-1), tartrate-resistant acid phosphatase 5b (TRAP-5b), and parathyroid hormone (PTH) in serum according to the manufacturers’ instructions. Hematoxylin and eosin (H&E) staining was performed on tissue sections of the small intestine, colon, pancreas, liver, distal femur, skeletal muscle, iWAT, and BAT to investigate tissue morphological changes between the Sham and OVX groups.

Mouse femoral specimens from six mice per group were fixed overnight in 4% paraformaldehyde (PFA) and scanned using a high-resolution micro-computed tomography (micro-CT) system (SkyScan 1272, Bruker, Germany). The scanning voltage and current were 60 kV and 170 μA, respectively; the scanning range was 2 cm × 2 cm, and the scanning resolution was 10 μm. Three-dimensional reconstruction was performed using NRecon, and bone volume fraction (BV/TV), trabecular thickness (Tb.Th), trabecular number (Tb.N), trabecular separation (Tb.Sp), and Cs.Th were analyzed using CTAn.

### 2.6 Shotgun metagenomic sequencing and analysis

Microbial DNA was extracted from colonic contents using the QIAamp DNA Stool Mini Kit (Qiagen) according to the manufacturer’s instructions. Extracts were treated with DNase-free RNase to remove RNA contamination, and DNA quantity and quality were assessed using a NanoDrop spectrophotometer, a Qubit Fluorometer with the Quant-iT dsDNA BR Assay Kit, and agarose gel electrophoresis. Paired-end libraries with an average insert size of 350 bp were constructed for each sample and sequenced on the BGI-SEQ500 platform to generate 2 × 150 bp reads. Raw data were filtered to remove adapters, low-quality reads, ambiguous bases, and host-derived contamination, yielding clean reads for downstream analysis. Across the 16 metagenomic libraries, the median number of quality-filtered reads was 40,804,098 per sample (range, 33,991,554–47,516,284), the median recorded host-sequence rate (Host_rate) was 5.41% (range, 0.59%–69.07%), and the median number of non-host clean reads retained for downstream analysis was 37,651,536 per sample (range, 12,532,396–45,089,622). All 16 samples were retained for downstream profiling.

Taxonomic profiling was performed using MetaPhlAn 4.0 with the mpa_vOct22_CHOCOPhlAnSGB_202212 marker database, and functional profiling was generated using HUMAnN 4.0. Alpha diversity was calculated from the taxonomic profiles in R (v4.0.3) using the vegan package, including richness (Number), Shannon index, Simpson index, and inverse Simpson index. Beta diversity was calculated on the basis of Bray–Curtis distances, and group-level community differences were evaluated by principal coordinates analysis (PCoA) and permutational multivariate analysis of variance (PERMANOVA). Differentially abundant taxa were identified using linear discriminant analysis (LDA) effect size (LEfSe), with a Kruskal–Wallis test alpha of 0.05 and an LDA score threshold of 2.0.

### 2.7 Transcriptome sequencing and analysis

Total RNA was isolated from colon, liver, and skeletal muscle tissues using TRIzol Reagent (Invitrogen). RNA concentration, purity, and integrity were evaluated using a NanoDrop spectrophotometer, and only samples with an RNA integrity number (RIN) ≥7.0 were used for library construction. mRNA-enriched transcriptome libraries were prepared using the TruSeq RNA Library Prep Kit v2 (Illumina) and sequenced on the NovaSeq 6000 platform (Illumina) to generate raw FASTQ files.

Raw reads were processed with Cutadapt (v1.15) to remove adapter sequences and low-quality bases. Clean reads were then aligned to the mouse reference genome using HISAT2 (v2.0.5). Gene-level read counts were generated with HTSeq (v0.9.1), and expression values were also normalized as fragments per kilobase of transcript per million mapped reads (FPKM). Differential expression analysis was performed on the gene-level raw count matrix using the DESeq2 R package (v1.30.0), with thresholds of absolute log2 fold change (|log2FC|) > 1 and *P* < 0.05. Bidirectional clustering and heatmap visualization of differentially expressed genes were performed using pheatmap (v1.0.8) in R based on Euclidean distance and complete linkage. Gene Ontology (GO) and Kyoto Encyclopedia of Genes and Genomes (KEGG) enrichment analyses of differentially expressed genes were conducted using clusterProfiler (v3.4.4), and gene set enrichment analysis (GSEA) was performed on ranked gene lists. Enrichment results with *P* < 0.05 were considered significant.

### 2.8 Untargeted metabolomic analysis

Biological samples were freeze-dried using a vacuum freeze-dryer (Scientz-100F) and pulverized in a mixer mill (MM 400, Retsch) with zirconia beads for 1.5 min at 30 Hz. Lyophilized powder (50 mg) was extracted in 1.2 mL of 70% methanol, vortexed repeatedly, and centrifuged at 12,000 rpm for 3 min. Supernatants were filtered through a 0.22-μm membrane (ANPEL) before UPLC-MS/MS analysis.

Untargeted metabolomic profiling was performed on a UPLC-ESI-MS/MS system consisting of a SHIMADZU Nexera X2 UPLC platform coupled to an Applied Biosystems 6500 QTRAP mass spectrometer. Chromatographic separation was achieved on an Agilent SB-C18 column (1.8 μm, 2.1 × 100 mm) with water containing 0.1% formic acid as solvent A and acetonitrile containing 0.1% formic acid as solvent B. The gradient was programmed from 95% A/5% B to 5% A/95% B over 9 min, held for 1 min, returned to 95% A/5% B within 1.1 min, and equilibrated for 2.9 min. The flow rate was 0.35 mL/min, the column temperature was 40 °C, and the injection volume was 2 μL. The ESI source was operated with a source temperature of 500 °C and ion spray voltages of 5,500 V in positive ion mode and −4,500 V in negative ion mode. Ion source gas I, gas II, and curtain gas were set at 50, 60, and 25 psi, respectively, and collision-activated dissociation was set to high. Multiple reaction monitoring (MRM) transitions were optimized for declustering potential and collision energy.

Unsupervised principal component analysis (PCA) was performed in R using unit variance-scaled data. Hierarchical cluster analysis (HCA) and Pearson correlation coefficient (PCC) analyses were performed using the ComplexHeatmap package. Differential metabolites between groups were selected using *P* < 0.05 and an absolute log2 fold change (|log2FC| ≥ 1.0). Variable importance in projection (VIP) scores derived from orthogonal partial least-squares discriminant analysis (OPLS-DA) models were used for feature ranking. OPLS-DA models, score plots, and permutation tests (200 permutations) were generated using MetaboAnalystR. Identified metabolites were annotated using the KEGG Compound and Pathway databases, and metabolite set enrichment analysis was performed using the hypergeometric test.

### 2.9 Targeted quantification of bile acids and short-chain fatty acids

For targeted bile acid (BA) analysis, individual BA standards were mixed in BA-free matrix to generate calibration curves, and isotope-labeled compounds were used as internal standards. Samples were diluted in water, mixed with acetonitrile/methanol (8:2) containing internal standards, sonicated for 10 min, incubated at −20 °C for 60 min, and centrifuged at 12,000 rpm for 10 min. Supernatants were analyzed on an ExionLC AD UHPLC-QTRAP 6500+ system (AB SCIEX) in Novogene Co., Ltd. Chromatographic separation was performed on a Waters ACQUITY UPLC BEH C18 column (2.1 × 100 mm, 1.7 μm) at 50 °C using 0.1% formic acid and 5 mM ammonium acetate in water as solvent A and acetonitrile as solvent B at a flow rate of 0.35 mL/min. The mass spectrometer was operated in negative MRM mode with an ion spray voltage of −4,500 V, curtain gas at 30 psi, ion source temperature of 550 °C, and ion source gases 1 and 2 at 60 psi.

For targeted short-chain fatty acid (SCFA) analysis, 11 SCFA standards were obtained from ZZ Standards Co., Ltd., and isotope-labeled standards were used as internal standards. SCFA quantification was performed on a Vanquish Flex UHPLC-TSQ Altis system (Thermo Scientific) in Novogene Co., Ltd. Separation was carried out on a Waters ACQUITY UPLC BEH C18 column (2.1 × 100 mm, 1.7 μm) maintained at 40 °C. The mobile phase consisted of 10 mM ammonium acetate in water as solvent A and acetonitrile:isopropanol (1:1) as solvent B, delivered at 0.30 mL/min. The mass spectrometer was operated in negative MRM mode with an ion spray voltage of −4,500 V, sheath gas at 35 psi, ion source temperature of 550 °C, auxiliary gas at 50 psi, and collision gas at 55 psi.

### 2.10 Statistical analysis

Statistical analyses were performed using R. Group comparisons for continuous variables were conducted using the Wilcoxon rank-sum test or Student’s *t*-test, as appropriate. The serum biomarkers and the quantitative micro-CT outcomes were evaluated using two-sided Wilcoxon rank-sum tests. For beta-diversity analysis, Bray–Curtis distances were used for ordination, and PERMANOVA was applied to evaluate overall group separation. Differential transcriptomic and metabolomic features were identified according to the thresholds described above. A two-sided *P* value < 0.05 was considered statistically significant. Associations between body weight and omics features and across omics layers were evaluated using Spearman correlation analysis with Sham and OVX mice analyzed together. Correlation coefficients and two-sided *P* values were reported.

## 3 Results

### 3.1 OVX-associated systemic phenotypic and biochemical differences in mice

**Figure 1:**
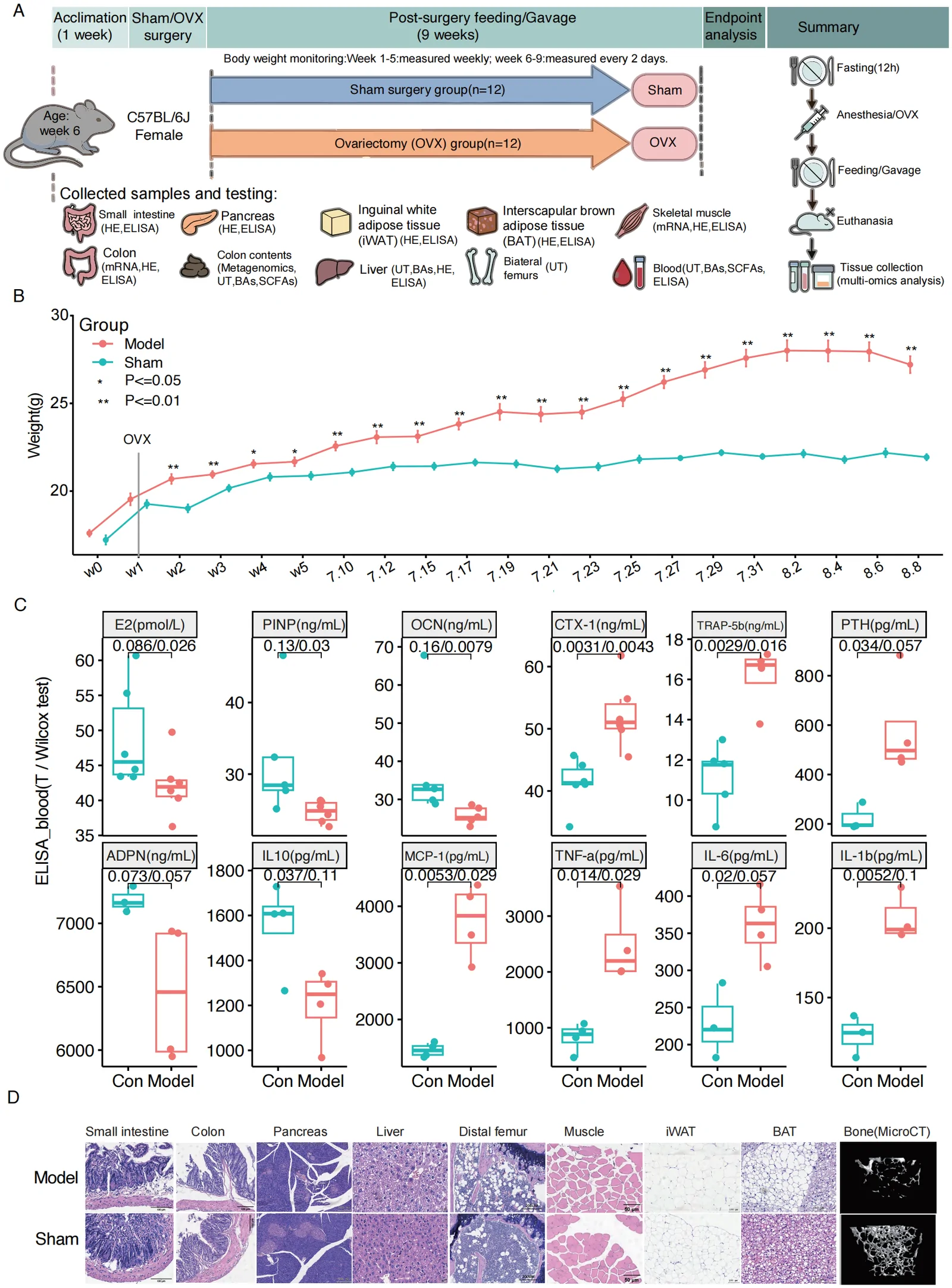
Experimental design, longitudinal body weight, serum biochemical measurements, multitissue histology, and representative femoral microcomputed tomography (micro-CT) findings in Sham and OVX mice. **(A)** Six-week-old female C57BL/6J mice were acclimated for 1 week and then underwent sham or bilateral ovariectomy (OVX) surgery (12 mice assigned per group), followed by 9 weeks of observation and endpoint sample collection after a 12-h fast. Body weight was recorded weekly during postoperative weeks 1–5 and every 2 days during weeks 6–9. **(B)** Longitudinal body-weight measurements before and after surgery; the vertical dashed line marks the surgery. *P ≤ 0.05 and **P ≤ 0.01. **(C)** Serum E2, PINP, OCN, CTX-1, TRAP-5b, PTH, ADPN, IL-10, MCP-1, TNF-α, IL-6, and IL-1β concentrations measured by ELISA. The two values above each plot are *P* values from Student’s *t*-test and the two-sided Wilcoxon rank-sum test, respectively; inferential statements in the text follow the Wilcoxon results. **(D)** Representative hematoxylin and eosin (H&E)-stained sections of the small intestine, colon, pancreas, liver, distal femur, skeletal muscle, inguinal white adipose tissue (iWAT), and interscapular brown adipose tissue (BAT), together with representative femoral micro-CT reconstructions. Scale bars: 100 μm for small intestine, colon, iWAT, and BAT; 200 μm for pancreas and distal femur; 20 μm for liver; and 50 μm for skeletal muscle. Con and Model denote Sham and OVX, respectively, in panels retaining the source labels.

To establish the OVX model and characterize the systemic phenotype, 6-week-old female mice were acclimatized for 1 week, underwent bilateral ovariectomy (OVX) or sham surgery (Sham) at 7 weeks of age, and were maintained for a further 9 weeks. At the endpoint, serum, colonic, small intestine, colon, pancreas, liver, and skeletal muscle contents, inguinal white adipose tissue (iWAT), interscapular brown adipose tissue (BAT), and bilateral femurs were collected for subsequent analyses (**Figure 1A**). Body weights were comparable between groups at baseline (*P* = 0.25). During postoperative follow-up, OVX mice had higher body weights than Sham mice at the time points marked in **Figure 1B**, including the final recorded time point (*P* < 0.05). Serum ELISA profiling showed significantly lower E2, PINP, and OCN concentrations and significantly higher MCP-1, TNF-α, CTX-1, and TRAP-5b concentrations in OVX mice than in Sham mice. IL-10 and adiponectin (ADPN) concentrations were numerically lower, whereas IL-6, IL-1β, and PTH concentrations were numerically higher, but these comparisons did not reach statistical significance by the Wilcoxon test (**Figure 1C**).

Representative H&E-stained sections showed morphological differences between the OVX and Sham groups across multiple tissues (**Figure 1D**). In the small intestine and colon, OVX mice showed more irregular villus or mucosal-fold morphology and altered glandular organization. Pancreatic acinar structures appeared less compact, with wider interstitial spaces. In the liver, hepatocyte arrangement was less orderly, with focal pale staining and vacuole-like changes. Distal femoral sections appeared to contain less trabecular bone and a larger marrow cavity. Skeletal muscle showed differences in myofiber arrangement and interfiber spacing, whereas iWAT and BAT showed enlarged lipid-containing structures and altered adipocyte or lipid-droplet morphology. These were qualitative morphological observations. Representative femoral micro-CT reconstructions are shown in **Figure 1D**. Quantitative femoral micro-CT analysis showed lower bone volume fraction (BV/TV), trabecular thickness (Tb.Th), trabecular number (Tb.N), and Cs.Th, together with greater trabecular separation (Tb.Sp), in OVX mice than in Sham mice (*P* = 0.015 for Tb.Th and *P* = 0.0022 for all other parameters, two-sided Wilcoxon rank-sum test; **Supplementary Figure 1**). OVX mice showed higher body weights at multiple postoperative time points, lower serum E2, differences in inflammation-related and bone-turnover markers, morphological differences across tissues, and altered femoral microarchitecture.

### 3.2 OVX-associated gut microbial community alterations in mice

**Figure 2:**
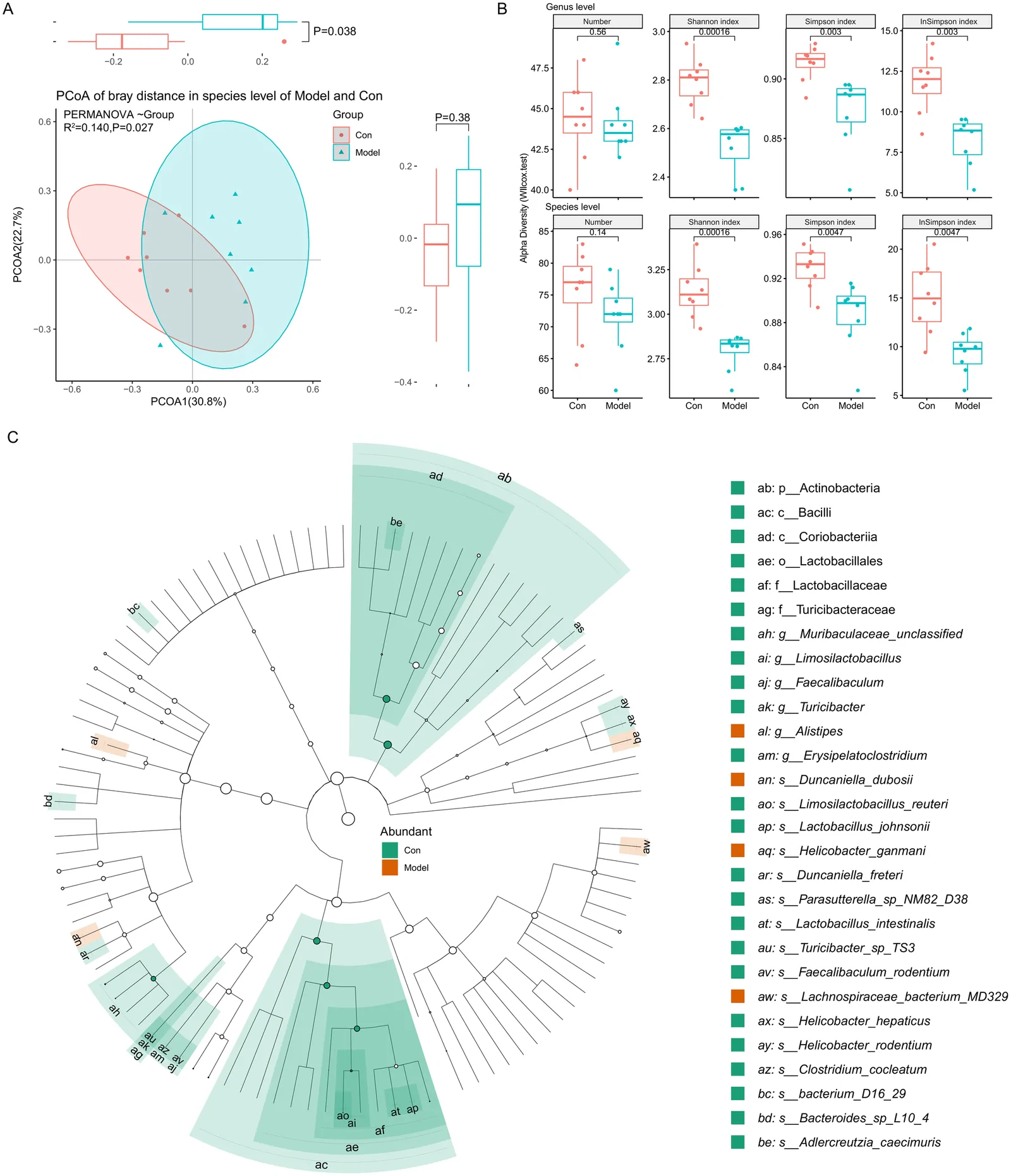
OVX-associated differences in the gut microbiota of colonic contents. **(A)** Species-level Bray–Curtis principal coordinates analysis (PCoA), with marginal boxplots showing the distributions of sample coordinates on the displayed axes. The overall group difference was evaluated by PERMANOVA (*R*² = 0.140, *P* = 0.027). **(B)** Alpha-diversity indices at the genus and species levels. Number denotes observed richness, or the number of detected taxa, whereas the Shannon, Simpson, and inverse Simpson indices incorporate the relative-abundance distribution and are therefore sensitive to community evenness and taxon dominance. **(C)** Linear discriminant analysis effect size (LEfSe) cladogram showing taxa that discriminated the Sham and OVX groups. *n* = 8 mice per group. Con denotes Sham and Model denotes OVX. LEfSe used *P* < 0.05 and an LDA score threshold of 2.0.

Shotgun metagenomic sequencing of colonic contents was used to compare gut microbial profiles between the OVX and Sham groups. Bray–Curtis-based PCoA showed significant overall separation between the OVX and Sham groups at both the species level (PERMANOVA, *R*² = 0.14, *P* = 0.027; **Figure 2A**) and the genus level (PERMANOVA, *R*² = 0.13, *P* = 0.045; **Supplementary Figure 2A**). By contrast, the overall UniRef functional-profile comparison was not significant (**Supplementary Figure 2D**). Shannon, Simpson, and inverse Simpson indices were lower in OVX mice at both the genus and species levels, whereas richness (Number) did not differ significantly (**Figure 2B**).

At the phylum level, Actinobacteria had a lower relative abundance in OVX mice. At the genus level, *Turicibacter*, *Faecalibaculum*, *Limosilactobacillus*, and unclassified Muribaculaceae had lower relative abundances. At the species level, *Lactobacillus johnsonii*, *Lactobacillus intestinalis*, *Limosilactobacillus reuteri*, and *Duncaniella freteri* had lower relative abundances. By contrast, *Helicobacter ganmani* and *Duncaniella dubosii* had higher relative abundances in OVX mice (**Supplementary Figure 3**). Linear discriminant analysis effect size (LEfSe) identified *Alistipes*, *Duncaniella dubosii*, *Helicobacter ganmani*, and Lachnospiraceae bacterium MD329 as representative OVX-enriched taxa. In contrast, Sham-enriched features included *Limosilactobacillus*, *Faecalibaculum*, *Turicibacter*, *Erysipelatoclostridium*, unclassified Muribaculaceae, *Lactobacillus johnsonii*, *Lactobacillus intestinalis*, *Limosilactobacillus reuteri*, *Duncaniella freteri*, *Faecalibaculum rodentium*, *Turicibacter* sp. TS3, *Parasutterella* sp. NM82_D38, *Helicobacter hepaticus*, *Helicobacter rodentium*, *Clostridium cocleatum*, *Bacteroides* sp. L10_4, and *Adlercreutzia caecimuris* (**Figure 2C**; **Supplementary Figures 3** and **4**). Sham and OVX mice were analyzed together using Spearman correlation analysis. *Turicibacter* relative abundance was inversely correlated with body weight (*ρ* = −0.782, *P* = 3.41 × 10⁻^4^). Body weight was also inversely correlated with the relative abundances of *Lactobacillus johnsonii* (*ρ* = −0.526, *P* = 0.0364), *Lactobacillus intestinalis* (*ρ* = −0.613, *P* = 0.0116), and *Limosilactobacillus reuteri* (*ρ* = −0.506, *P* = 0.0456; **Supplementary Figure 5**; **Supplementary Table 1**).

### 3.3 OVX-associated metabolomic alterations in colonic contents

**Figure 3:**
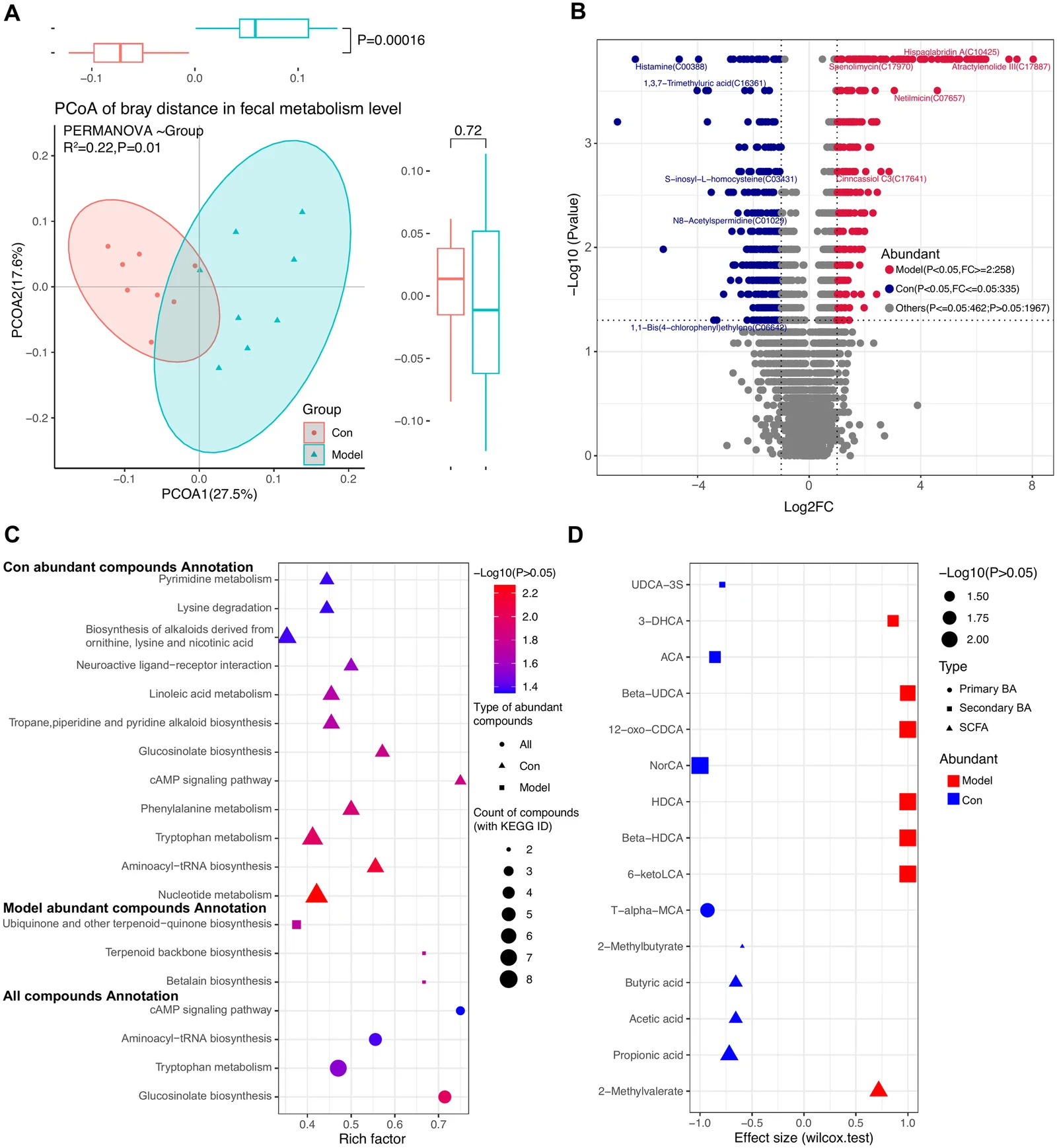
OVX-associated differences in the colonic-content metabolome. **(A)** Bray–Curtis-based PCoA, with marginal boxplots showing the distributions of sample coordinates on the displayed axes. The overall group difference was evaluated by PERMANOVA (*R*² = 0.22, *P* = 0.01). **(B)** Volcano plot of differential metabolite features using the screening criteria shown in the panel; 258 features were more abundant in OVX and 335 were more abundant in Sham. **(C)** Kyoto Encyclopedia of Genes and Genomes (KEGG) annotation of all annotated metabolites and of metabolites more abundant in each group. **(D)** Effect-size plot of differential short-chain fatty acids (SCFAs) and bile acids (BAs). Significance encodings should be read as −log10(*P* value). *n* = 8 mice per group. Con denotes Sham and Model denotes OVX.

The colonic-content metabolome was compared between OVX and Sham mice. Bray–Curtis-based PCoA showed a significant overall difference between the groups (PERMANOVA, *R*² = 0.22, *P* = 0.01), primarily along PCoA1 (*P* = 0.00016), whereas PCoA2 did not differ significantly (*P* = 0.72; **Figure 3A**). Using the fold-change (FC) thresholds FC ≥ 2 or FC ≤ 0.5 and *P* < 0.05, differential analysis identified 258 metabolite features with higher relative abundance in OVX and 335 with higher relative abundance in Sham. Histamine and 5-hydroxyindole-3-acetic acid (5-HIAA) had lower relative abundances in OVX mice (**Figure 3B**).

KEGG annotations included tryptophan metabolism, aminoacyl-tRNA biosynthesis, glucosinolate biosynthesis, and cAMP signaling. Sham-higher features were mainly annotated to pyrimidine metabolism, lysine degradation, linoleic acid metabolism, phenylalanine metabolism, tryptophan metabolism, aminoacyl-tRNA biosynthesis, nucleotide metabolism, cAMP signaling, neuroactive ligand–receptor interaction, and several alkaloid-biosynthesis pathways. In contrast, OVX-higher features were mainly annotated to ubiquinone and other terpenoid–quinone biosynthesis, terpenoid-backbone biosynthesis, and betalain biosynthesis (**Figure 3C**). Propionic acid, acetic acid, butyric acid, and 2-methylbutyrate were lower in OVX mice, whereas 2-methylvalerate was higher (*P* < 0.05; **Figure 3D**). The bile-acid profile also differed, with UDCA-3S, ACA, NorCA, and T-α-MCA higher in Sham and 3-DHCA, β-UDCA, 12-oxo-CDCA, HDCA, β-HDCA, and 6-ketoLCA higher in OVX (*P* < 0.05; **Figure 3D**). OPLS-DA highlighted L-tryptophan, serotonin, N-acetylserotonin, 5-hydroxyindole-3-acetic acid, 3-methylindole, and linoleic acid among the annotated discriminant features (**Supplementary Figures 6**, **7**).

Spearman correlations were subsequently examined with data from Sham and OVX mice analyzed together. Positive correlations were observed between Actinobacteria relative abundance and N-acetylserotonin relative abundance (*ρ* = 0.918, *P* = 5.53 × 10⁻^7^), between *Erysipelatoclostridium* relative abundance and 3-methylindole relative abundance (*ρ* = 0.897, *P* = 2.58 × 10⁻^6^), and between *Turicibacter* relative abundance and histamine relative abundance (*ρ* = 0.879, *P* = 7.19 × 10⁻^6^). *Duncaniella dubosii* relative abundance was inversely correlated with serotonin relative abundance (*ρ* = −0.697, *P* = 0.00269), and *Helicobacter ganmani* relative abundance was inversely correlated with histamine relative abundance (*ρ* = −0.654, *P* = 0.00601). Body weight was positively correlated with diosgenin (*ρ* = 0.656, *P* = 0.00580) and prephenic acid (*ρ* = 0.635, *P* = 0.00818) relative abundances and inversely correlated with histamine (*ρ* = −0.776, *P* = 4.04 × 10⁻^4^) and lysophosphatidylcholine (LPC) 18:2 (*ρ* = −0.818, *P* = 1.09 × 10⁻^4^) relative abundances (**Supplementary Figure 8**).

### 3.4 OVX-associated colonic transcriptomic alterations in mice

**Figure 4:**
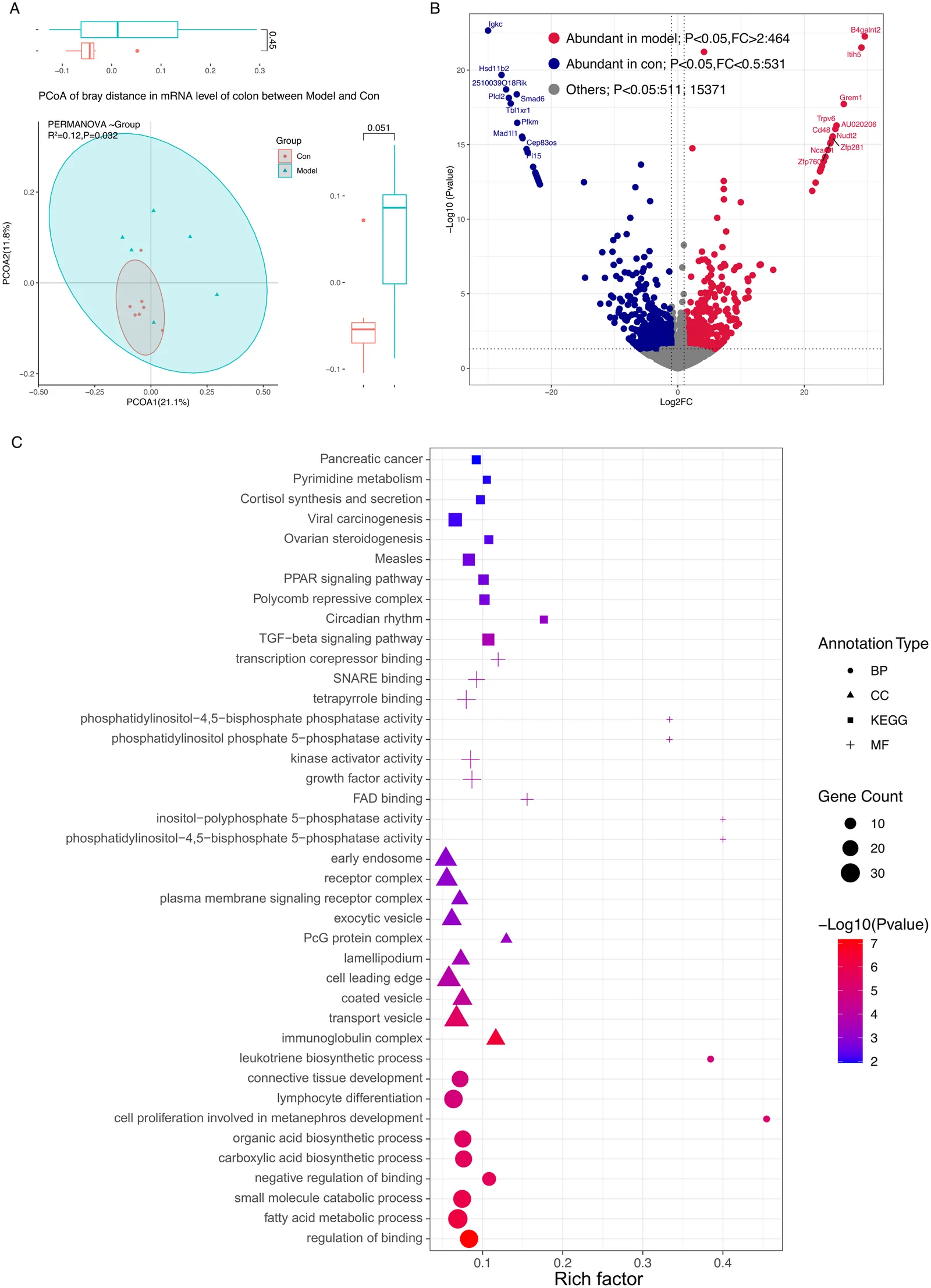
OVX-associated differences in the colonic transcriptome. **(A)** Bray–Curtis-based PCoA, with marginal boxplots showing the distributions of sample coordinates on the displayed axes. The overall group difference was significant by PERMANOVA (*R*² = 0.12, *P* = 0.032), whereas the axis-wise comparisons were not significant for either PCoA1 (*P* = 0.45) or PCoA2 (*P* = 0.051). **(B)** Volcano plot of genes meeting the displayed screening thresholds (|log2FC| > 1 and nominal *P* < 0.05); 464 genes had higher and 531 had lower expression in OVX mice. **(C)** Gene Ontology (GO) and KEGG enrichment analysis of genes meeting these screening thresholds. *n* = 13 mice (7 Sham and 6 OVX). Con denotes Sham, and Model denotes OVX.

Colonic transcriptomic profiling was used to examine gene expression in colon tissue. Bray–Curtis-based PCoA showed a significant overall difference between the OVX and Sham groups by PERMANOVA (*R*² = 0.12, *P* = 0.032), whereas the axis-wise comparisons were not significant for either PCoA1 (*P* = 0.45) or PCoA2 (*P* = 0.051; **Figure 4A**). DESeq2 was used to identify differentially expressed genes (DEGs) between the OVX and Sham groups, with thresholds of |log2 fold change| > 1 and *P* < 0.05. Of these, 464 genes exhibited higher expression, whereas 531 genes showed a lower expression in OVX mice. Representative OVX-higher genes included *Itih5*, *Grem1*, *Trpv6*, *B4galnt2*, *Nudt2*, *Cd48*, and *Ncam1*, whereas representative Sham-higher genes included *Igkc*, *Hsd11b2*, *Plcl2*, *Smad6*, *Pfkm*, and *Pi15* (**Figure 4B**).

GO enrichment analysis identified functional terms across the three ontology domains. At the biological-process level, representative terms included fatty acid metabolic process, small-molecule catabolic process, organic- and carboxylic-acid biosynthetic processes, leukotriene biosynthetic process, lymphocyte differentiation, connective-tissue development, and regulation of binding. At the cellular-component level, representative terms included the immunoglobulin complex, transport vesicle, coated vesicle, exocytic vesicle, early endosome, receptor complex, plasma-membrane signaling receptor complex, lamellipodium, and cell leading edge. At the molecular-function level, representative terms included transcription corepressor binding, SNARE binding, tetrapyrrole binding, phosphatidylinositol-phosphatase activities, kinase activator activity, growth factor activity, and FAD binding. KEGG annotations included TGF-β and PPAR signaling, ovarian steroidogenesis, cortisol synthesis and secretion, circadian rhythm, and pyrimidine metabolism (**Figure 4C**). Ranked-list GSEA identified Sham-enriched gene sets associated with the basolateral plasma membrane, brush border, endoplasmic-reticulum protein-containing complex, protein folding, oxidoreductase and protein-disulfide reductase activities, and regulation of protein localization to the cell periphery and plasma membrane (**Supplementary Figure 9**). The displayed KEGG gene sets were also Sham-enriched and included AMP-activated protein kinase (AMPK), cGMP–PKG, and estrogen signaling; bile secretion; carbon metabolism; bacterial invasion of epithelial cells; gastric acid secretion; aldosterone synthesis and secretion; thyroid hormone synthesis; and protein processing in the endoplasmic reticulum (**Supplementary Figure 10**). The enriched terms were related to epithelial polarity and brush-border organization, vesicle-associated processes, endoplasmic-reticulum processing, and metabolic and energy-sensing pathways. Among genes associated with these pathways, *Pck1*, *Acsl1*, and *Hsd3b3* had lower expression in OVX mice, whereas *Dbp* had higher expression.

Spearman correlation analysis was applied to the combined dataset from Sham and OVX mice. Body weight was positively correlated with *Nr1d1* (*ρ* = 0.769, *P* = 0.00211), *Dbp* (*ρ* = 0.835, *P* = 3.80 × 10⁻^4^), and *Per2* (*ρ* = 0.835, *P* = 3.80 × 10⁻^4^) expression and inversely correlated with *Bmp8a* expression (*ρ* = −0.695, *P* = 0.00834). Positive correlations were observed between *Helicobacter rodentium* relative abundance and *Kcnk2* expression (*ρ* = 0.979, *P* = 6.76 × 10⁻^9^), *Turicibacter* relative abundance and *Pck1* expression (*ρ* = 0.830, *P* = 4.50 × 10⁻^4^), and *Duncaniella freteri* relative abundance and *Acsl1* expression (*ρ* = 0.834, *P* = 3.91 × 10⁻^4^). Positive correlations were also observed for *Duncaniella dubosii*–*Dbp* (*ρ* = 0.687, *P* = 0.00951), *Turicibacter*–*Hsd3b3* (*ρ* = 0.703, *P* = 0.00732), *Duncaniella dubosii*–*Nr1d1* (*ρ* = 0.725, *P* = 0.00502), and *Helicobacter ganmani*–*Dbp* (*ρ* = 0.650, *P* = 0.01618). Inverse correlations were observed for *Lactobacillus johnsonii*–*Nr1d1* (*ρ* = −0.691, *P* = 0.00898), *Limosilactobacillus reuteri*–*Dbp* (*ρ* = −0.846, *P* = 2.66 × 10⁻^4^), and *Alistipes*–*Acsl1* (*ρ* = −0.746, *P* = 0.00342; **Supplementary Figure 11**).

### 3.5 OVX-associated serum bile acid and short-chain fatty acid alterations in mice

**Figure 5:**
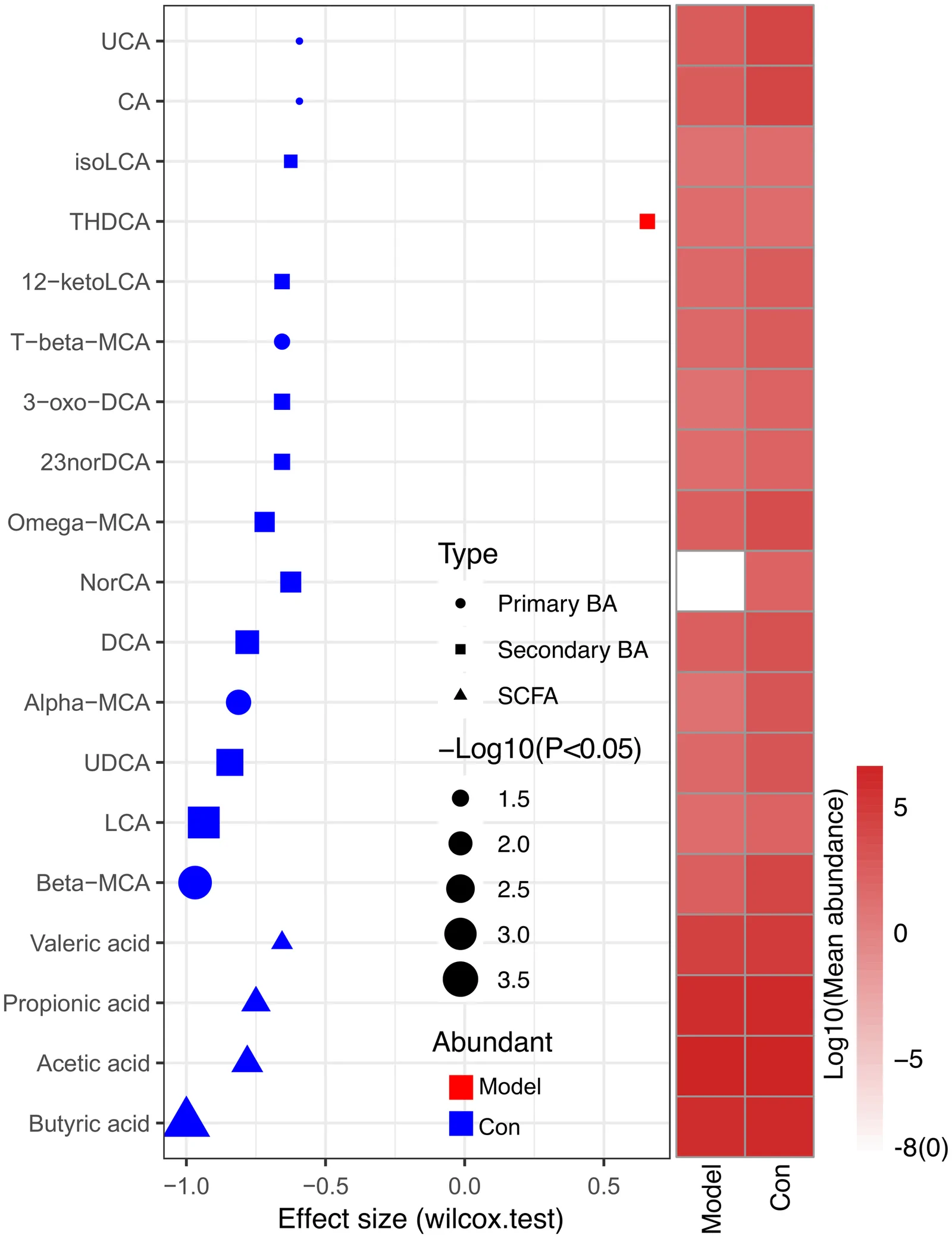
Serum bile acid and short-chain fatty acid differences between Sham and OVX mice. The left panel ranks 19 differential analytes by effect size; point shape denotes metabolite class, and color denotes the group with the higher concentration. A total of 15 analytes were BAs, and 4 were SCFAs; 18 had lower concentrations in OVX, whereas THDCA had a higher concentration. The right panel shows log10-transformed group means for the corresponding analytes. The size key should be read as −log10(*P* value). *n* = 8 mice per group. Con denotes Sham and Model denotes OVX. Between-group comparisons were performed as described in Methods; *P* < 0.05 was considered statistically significant.

Targeted serum bile acid and short-chain fatty acid profiling identified 19 analytes that differed between Sham and OVX mice, 18 of which had lower concentrations in OVX mice. Bile acids accounted for 15 of these analytes. UCA, CA, isoLCA, 12-ketoLCA, T-β-MCA, 3-oxo-DCA, 23norDCA, ω-MCA, NorCA, DCA, α-MCA, UDCA, LCA, and β-MCA concentrations were lower in OVX mice, whereas THDCA was the only analyte with a higher concentration. Serum valerate, propionate, acetate, and butyrate concentrations were also lower in OVX mice (*P* < 0.05; **Figure 5**). Spearman analysis across the two groups was used to assess associations involving body weight, gut taxa, and circulating metabolites. Body weight was positively correlated with serum THDCA concentration (*ρ* = 0.659, *P* = 0.00546) and inversely correlated with serum β-MCA (*ρ* = −0.703, *P* = 0.00239) and butyrate (*ρ* = −0.688, *P* = 0.00320) concentrations. *Turicibacter* relative abundance was positively correlated with serum butyrate concentration (*ρ* = 0.653, *P* = 0.00610), *Lactobacillus johnsonii* relative abundance was positively correlated with serum butyrate concentration (*ρ* = 0.630, *P* = 0.00895), and *Lactobacillus intestinalis* relative abundance was positively correlated with serum β-MCA concentration (*ρ* = 0.655, *P* = 0.00589). By contrast, *Alistipes* relative abundance was inversely correlated with serum acetate concentration (*ρ* = −0.644, *P* = 0.00708), and *Duncaniella dubosii* relative abundance was inversely correlated with serum T-β-MCA concentration (*ρ* = −0.756, *P* = 7.06 × 10⁻^4^; **Supplementary Figure 12**).

### 3.6 OVX-associated hepatic metabolomic alterations in mice

**Figure 6:**
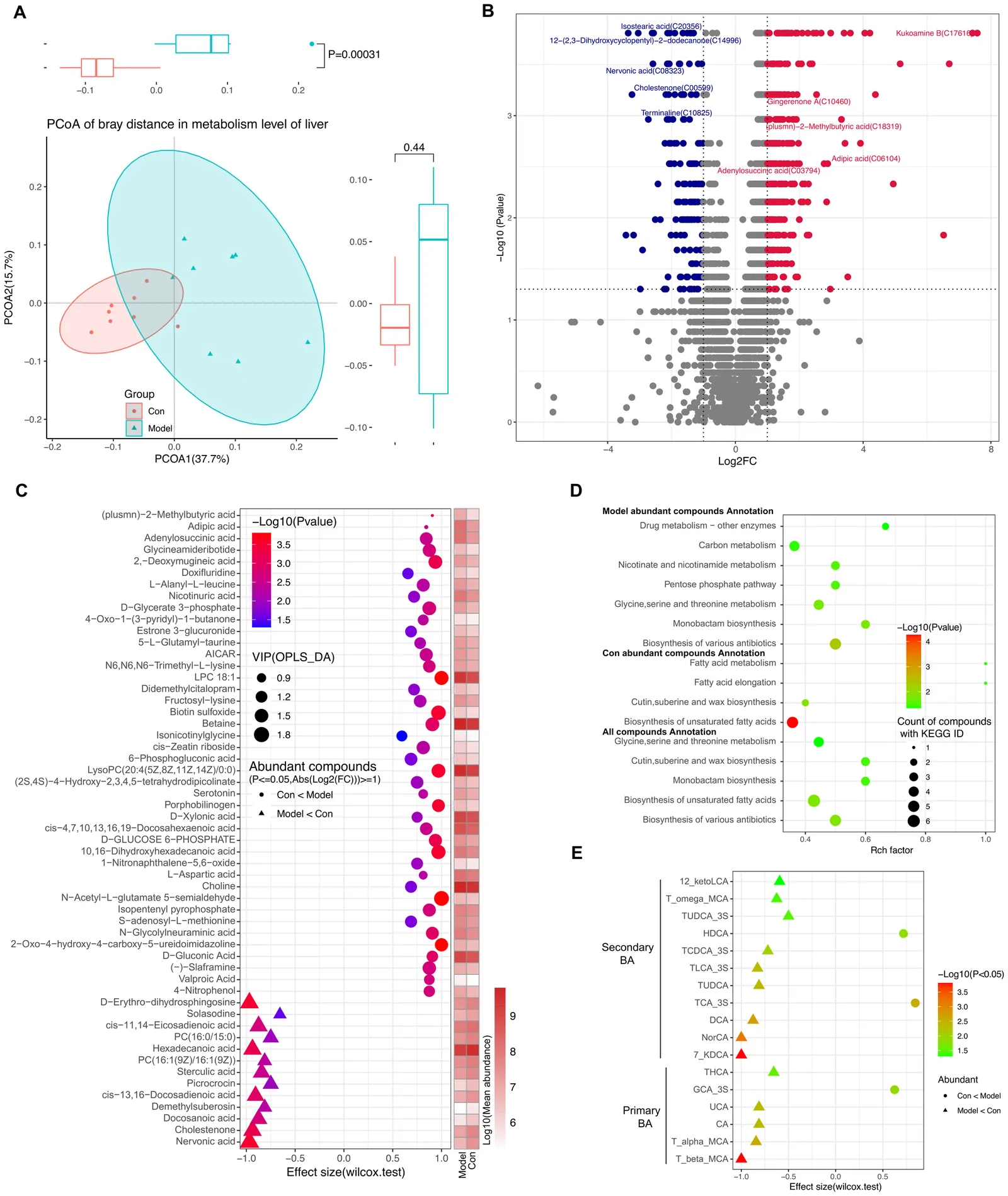
OVX-associated differences in the hepatic metabolome. **(A)** Bray–Curtis-based PCoA, with marginal boxplots showing sample-coordinate distributions. PCoA1 accounted for 37.7% of variation and differed between groups (*P* = 0.00031), whereas PCoA2 accounted for 15.7% and did not differ (*P* = 0.44). **(B)** Volcano plot of differential hepatic metabolite features. **(C)** Effect-size ranking and mean-abundance heatmap of differential metabolites. **(D)** KEGG annotation of all annotated metabolites and of metabolites more abundant in each group; the horizontal axis denotes the rich factor. **(E)** A total of 17 differential hepatic BAs, of which 14 had lower and 3 had higher contents in OVX mice; BA contents are reported in ng/g. Significance encodings should be read as −log10(*P* value). *n* = 8 mice per group. Con denotes Sham, and Model denotes OVX.

Hepatic metabolomic profiling showed a group difference along PCoA1 (37.7% of the variance; *P* = 0.00031), whereas PCoA2 accounted for 15.7% of the variance and PCoA2 scores did not differ between groups (*P* = 0.44; **Figure 6A**). Metabolites with higher relative abundance in OVX mice included Kukoamine B, Gingerenone A, 2-methylbutyric acid, adipic acid, and adenylosuccinic acid, whereas isostearic acid, nervonic acid, cholestenone, terminaline, and 12-(2,3-dihydroxycyclopentyl)-2-dodecanone had higher relative abundance in Sham mice (**Figures 6B, C**). KEGG annotation linked metabolites higher in OVX mice mainly to carbon metabolism, nicotinate and nicotinamide metabolism, the pentose phosphate pathway, and glycine, serine, and threonine metabolism. In contrast, metabolites higher in Sham mice were annotated to fatty acid metabolism, fatty acid elongation, and the biosynthesis of unsaturated fatty acids (**Figure 6D**). The intrahepatic bile-acid profile also differed between groups. Among the 17 differential hepatic BAs, 14 had lower contents in OVX mice: 12-ketoLCA, T-ω-MCA, TUDCA-3S, TCDCA-3S, TLCA-3S, TUDCA, DCA, NorCA, 7-KDCA, THCA, UCA, CA, T-α-MCA, and T-β-MCA. Only HDCA, TCA-3S, and GCA-3S had higher contents (ng/g; **Figure 6E**).

For the hepatic metabolomic layer, Spearman correlations were calculated using data from Sham and OVX mice together. Body weight was positively correlated with hepatic L-tryptophan (*ρ* = 0.656, *P* = 0.00580) and indole (*ρ* = 0.706, *P* = 0.00225) relative abundances and inversely correlated with hepatic T-β-MCA (*ρ* = −0.729, *P* = 0.00134) and DCA (*ρ* = −0.674, *P* = 0.00423) contents. An inverse correlation was observed between *Erysipelatoclostridium* relative abundance and hepatic D-gluconic acid relative abundance (*ρ* = −0.906, *P* = 1.38 × 10⁻^6^). Positive correlations were observed between *Adlercreutzia caecimuris* relative abundance and cholestenone relative abundance (*ρ* = 0.838, *P* = 5.01 × 10⁻^5^) and between *Faecalibaculum* relative abundance and UCA content (*ρ* = 0.800, *P* = 1.99 × 10⁻^4^). *Alistipes* relative abundance was positively correlated with hepatic indole relative abundance (*ρ* = 0.632, *P* = 0.00858), whereas *Turicibacter* relative abundance was inversely correlated with hepatic L-tryptophan relative abundance (*ρ* = −0.706, *P* = 0.00225) and *Helicobacter ganmani* relative abundance was inversely correlated with hepatic cholestenone relative abundance (*ρ* = −0.767, *P* = 5.23 × 10⁻^4^; **Supplementary Figure 13**).

### 3.7 OVX-associated hepatic transcriptomic alterations in mice

**Figure 7:**
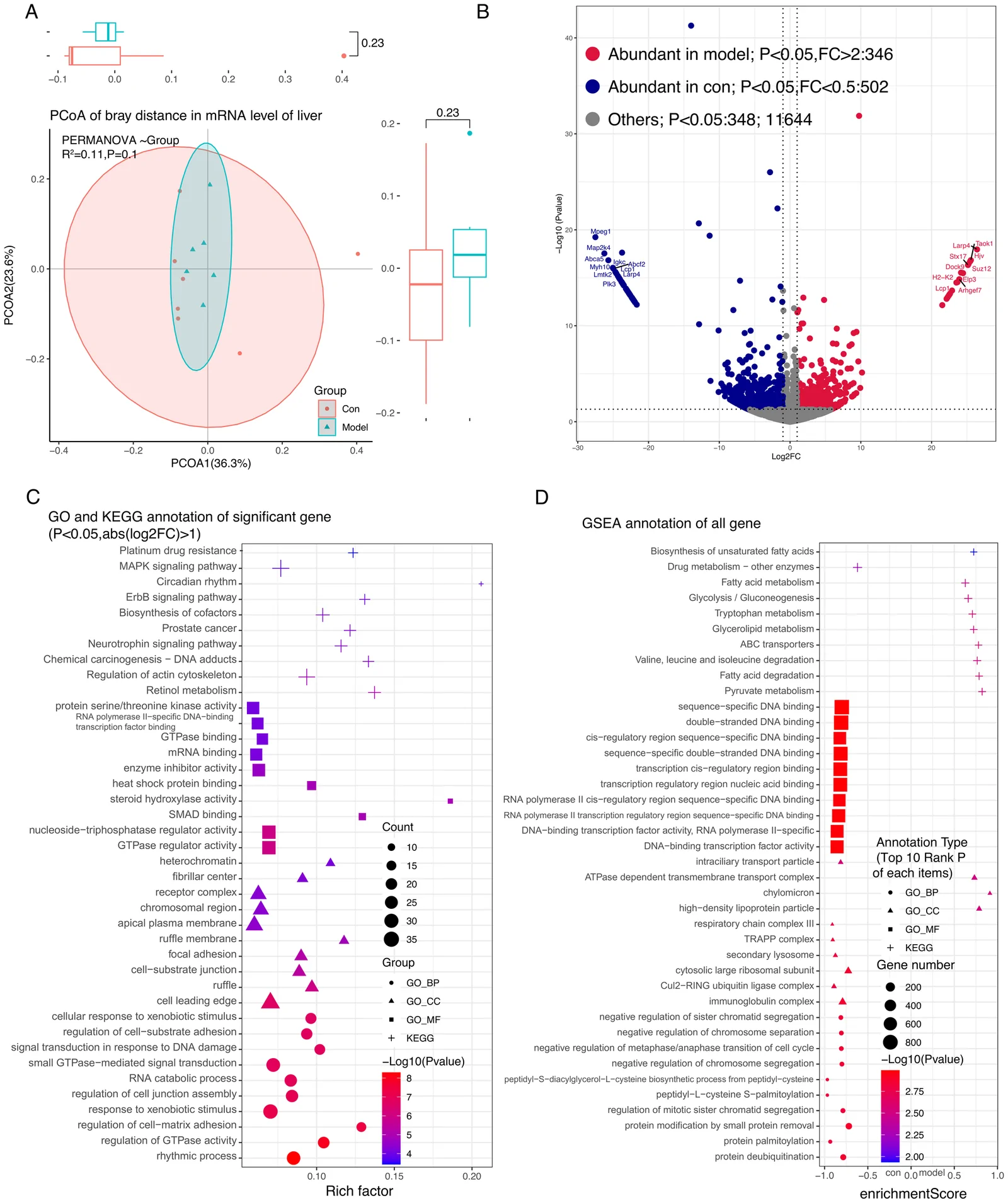
OVX-associated hepatic transcriptomic patterns. **(A)** Bray–Curtis-based PCoA, with marginal boxplots showing sample-coordinate distributions. The global PERMANOVA was not significant (*R*² = 0.11, *P* = 0.10), and both displayed axis-wise comparisons had *P* = 0.23. **(B)** Volcano plot of genes meeting the displayed screening thresholds (|log2FC| > 1 and nominal *P* < 0.05); 346 genes had higher and 502 had lower expression in OVX mice. **(C)** GO and KEGG enrichment analyses of genes meeting these thresholds. **(D)** Gene set enrichment analysis (GSEA) based on ranked gene lists. *n* = 13 mice (7 Sham and 6 OVX). Con denotes Sham, and Model denotes OVX. Gene-level and enrichment results are interpreted in the context of the global comparison.

Hepatic transcriptomic profiles did not show a statistically detectable global difference between groups (PERMANOVA, *R*² = 0.11, *P* = 0.10). PCoA1 and PCoA2 accounted for 36.3% and 23.6% of the variance, respectively, and scores on neither axis differed between groups (both *P* = 0.23; **Figure 7A**). Under the prespecified screening thresholds (|log2FC| > 1 and *P* < 0.05), 346 genes showed higher and 502 genes showed a lower expression in OVX mice. Representative OVX-higher genes included *Taok1*, *Hjv*, *Stx17*, *Dock9*, *H2-K2*, *Elp3*, and *Arhgef7*, whereas representative Sham-higher genes included *Mpeg1*, *Map2k4*, *Abca5*, *Myh10*, and *Igkc* (**Figure 7B**). GO and KEGG enrichment analyses identified annotations among the differentially expressed genes. At the biological-process level, annotations included rhythmic processes, regulation of GTPase activity, cell–matrix adhesion, xenobiotic responses, cell-junction assembly, RNA catabolism, and small-GTPase-mediated signal transduction. Molecular-function terms included steroid hydroxylase activity, heat-shock protein binding, SMAD binding, GTPase regulator activity, nucleoside-triphosphatase regulator activity, and protein serine/threonine kinase activity. Corresponding cellular-component terms included focal adhesion, cell–substrate junction, apical plasma membrane, receptor complex, cell leading edge, and ruffle membrane. KEGG analysis highlighted circadian rhythm, retinol metabolism, regulation of the actin cytoskeleton, MAPK signaling, biosynthesis of cofactors, and neurotrophin signaling (**Figure 7C**). GSEA identified gene sets enriched in each group. Sham-enriched gene sets included protein deubiquitination and protein palmitoylation. Other Sham-enriched gene sets involved ubiquitin-ligase, ribosomal, lysosomal, and intracellular-transport components. In contrast, OVX-enriched gene sets included high-density lipoprotein particles, chylomicrons, ATPase-dependent transport, biosynthesis of unsaturated fatty acids, fatty acid metabolism, glycolysis/gluconeogenesis, tryptophan and glycerolipid metabolism, ATP-binding cassette (ABC) transporters, branched-chain amino-acid degradation, and pyruvate metabolism (**Figure 7D**). The enriched gene sets involved protein processing, lipid handling, energy metabolism, and molecular transport. Hepatic transcriptomic associations were evaluated by Spearman analysis across both experimental groups. Body weight was positively correlated with hepatic *Per2* (*ρ* = 0.676, *P* = 0.01117), *Ak2* (*ρ* = 0.742, *P* = 0.00370), and *Cxcr4* (*ρ* = 0.769, *P* = 0.00211) expression and inversely correlated with *Rdh11* (*ρ* = −0.780, *P* = 0.00165) and *Cyp3a41a* (*ρ* = −0.668, *P* = 0.01267) expression. An inverse correlation was observed between Actinobacteria relative abundance and *Bhlhe40* expression (*ρ* = −0.775, *P* = 0.00187). Positive correlations were observed between *Erysipelatoclostridium* relative abundance and *Cdkn1a* expression (*ρ* = 0.859, *P* = 1.66 × 10⁻^4^), *Lactobacillus johnsonii* relative abundance and *Kynu* expression (*ρ* = 0.783, *P* = 0.00156), and *Lactobacillus intestinalis* relative abundance and *Cyp3a41a* expression (*ρ* = 0.719, *P* = 0.00563; **Supplementary Figure 14**).

### 3.8 OVX-associated skeletal muscle transcriptomic alterations in mice

**Figure 8:**
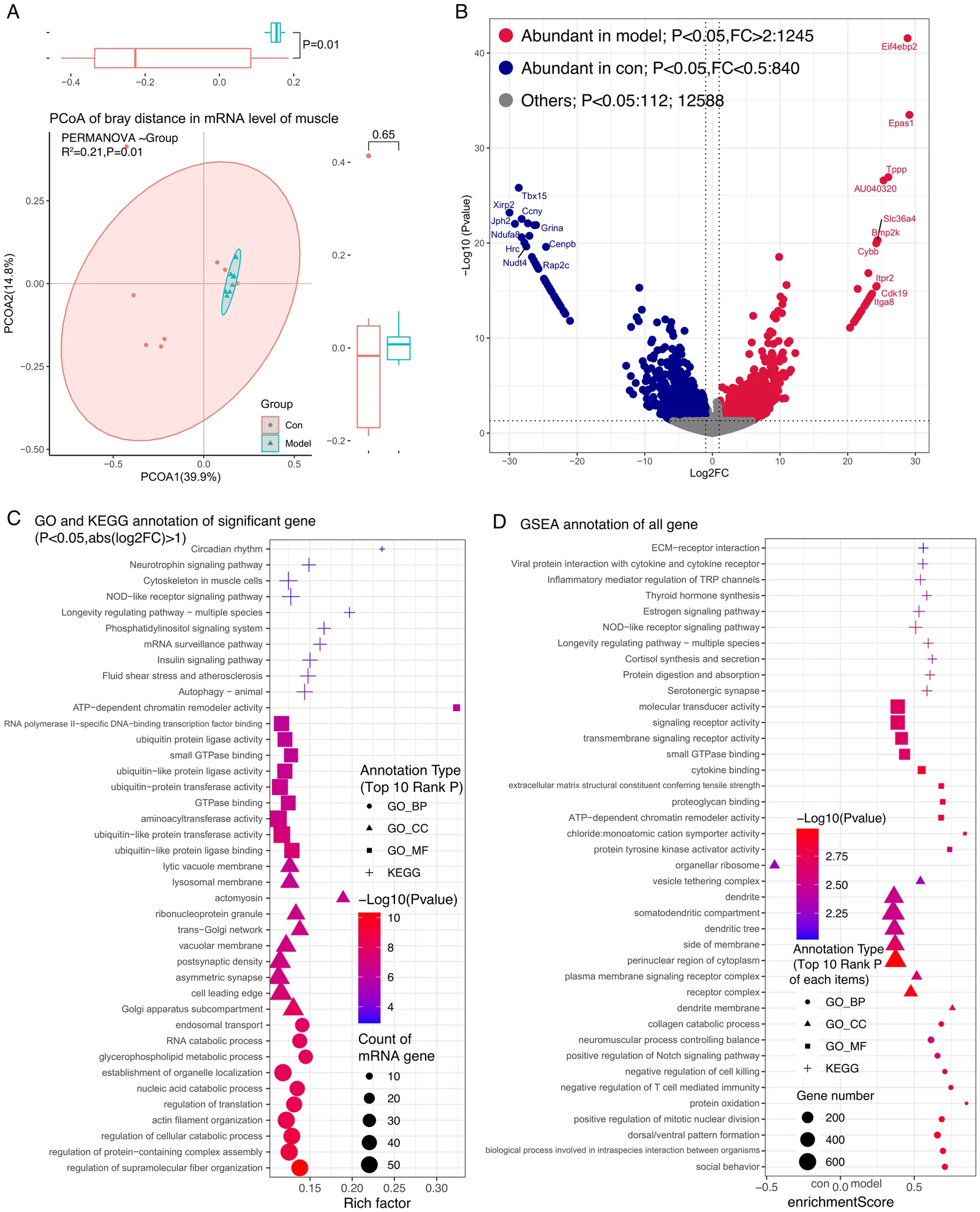
OVX-associated differences in the skeletal-muscle transcriptome. **(A)** Bray–Curtis-based PCoA, with marginal boxplots showing sample-coordinate distributions. The overall group difference was significant by PERMANOVA (*R*² = 0.21, *P* = 0.01). **(B)** Volcano plot of genes meeting the displayed screening thresholds (|log2FC| > 1 and nominal *P* < 0.05); 1,245 genes had a higher and 840 had a lower expression in OVX mice. **(C)** GO and KEGG enrichment analyses of genes meeting these thresholds. **(D)** GSEA based on ranked gene lists. Enrichment terms denote gene-set annotations rather than direct measurements of functional activity. *n* = 16 mice (8 Sham and 8 OVX). Con denotes Sham and Model denotes OVX.

In contrast to the hepatic transcriptome, skeletal muscle transcriptomic profiles differed significantly between the OVX and Sham groups at the global level (PERMANOVA, *R*² = 0.21, *P* = 0.01). PCoA1 and PCoA2 accounted for 39.9% and 14.8% of the variance, respectively. PCoA1 scores differed between groups (*P* = 0.01), whereas PCoA2 scores did not (*P* = 0.65; **Figure 8A**). Differential expression analysis using the prespecified thresholds (|log2FC| > 1 and *P* < 0.05) identified 1,245 genes with higher and 840 genes with a lower expression in OVX mice. Representative OVX-higher genes included *Eif4ebp2*, *Epas1*, *Tppp*, *Slc36a4*, *Cybb*, *Itpr2*, and *Itga8*, whereas representative Sham-higher genes included *Xirp2*, *Tbx15*, *Jph2*, *Ccny*, *Ndufa8*, and *Hrc* (**Figure 8B**). GO annotation of the DEGs showed that, at the cellular-component level, representative terms included lytic vacuole membrane, lysosomal membrane, vacuolar membrane, actomyosin, ribonucleoprotein granule, trans-Golgi network, cell leading edge, and Golgi apparatus subcompartment. Biological-process terms included endosomal transport, RNA catabolic process, glycerophospholipid metabolic process, establishment of organelle localization, regulation of translation, actin-filament organization, regulation of protein-containing complex assembly, and regulation of supramolecular-fiber organization. Molecular-function terms included ATP-dependent chromatin remodeler activity, RNA polymerase II-specific DNA-binding transcription factor binding, ubiquitin-protein ligase activity, ubiquitin-protein transferase activity, small-GTPase binding, and aminoacyltransferase activity. KEGG pathway annotation included circadian rhythm, neurotrophin signaling pathway, cytoskeleton in muscle cells, nucleotide-binding oligomerization domain (NOD)-like receptor signaling pathway, phosphatidylinositol signaling system, insulin signaling pathway, autophagy–animal, and mRNA surveillance pathway (**Figure 8C**). GSEA identified gene sets enriched in the OVX group, including extracellular matrix (ECM)–receptor interaction, inflammatory mediator regulation of transient receptor potential (TRP) channels, estrogen signaling pathway, NOD-like receptor signaling pathway, receptor and transmembrane-receptor activity, cytokine binding, extracellular-matrix-related terms, vesicle tethering complex, receptor complex, and other membrane-related complexes. Among the displayed terms, only the organellar ribosome gene set was enriched in the Sham group (**Figure 8D**). The enriched gene sets involved extracellular-matrix organization, membrane and receptor signaling, immune-related processes, and cellular structural regulation. In skeletal muscle, Spearman correlations were evaluated with Sham and OVX mice considered together. Body weight was positively correlated with the expression of *Ulk1* (*ρ* = 0.560, *P* = 0.02393), *Ern1* (*ρ* = 0.797, *P* = 2.17 × 10⁻^4^), and *Prkag3* (*ρ* = 0.595, *P* = 0.01497) in skeletal muscle and inversely correlated with *Ank2* expression (*ρ* = −0.788, *P* = 2.86 × 10⁻^4^). Inverse correlations were observed between *Duncaniella freteri* relative abundance and *Plcb4* expression (*ρ* = −0.922, *P* = 3.66 × 10⁻^7^), *Erysipelatoclostridium* relative abundance and *Cry2* expression (*ρ* = −0.863, *P* = 1.68 × 10⁻^5^), and *Turicibacter* relative abundance and *Prkag3* expression (*ρ* = −0.855, *P* = 2.50 × 10⁻^5^). Positive correlations were observed for *Alistipes*–*Acacb* (*ρ* = 0.703, *P* = 0.00238), *Duncaniella dubosii*–*Atg4c* (*ρ* = 0.642, *P* = 0.00732), and *Helicobacter ganmani*–*Myd88* (*ρ* = 0.677, *P* = 0.00399). Inverse correlations were observed for *Turicibacter*–*Atg2a* (*ρ* = −0.769, *P* = 5.04 × 10⁻^4^), *Lactobacillus johnsonii*–*Myd88* (*ρ* = −0.676, *P* = 0.00403), *Lactobacillus intestinalis*–*Ern1* (*ρ* = −0.698, *P* = 0.00266), and *Limosilactobacillus reuteri*–*Prkag3* (*ρ* = −0.671, *P* = 0.00446; **Supplementary Figure 15**).

### 3.9 OVX-associated bone metabolomic alterations in mice

**Figure 9:**
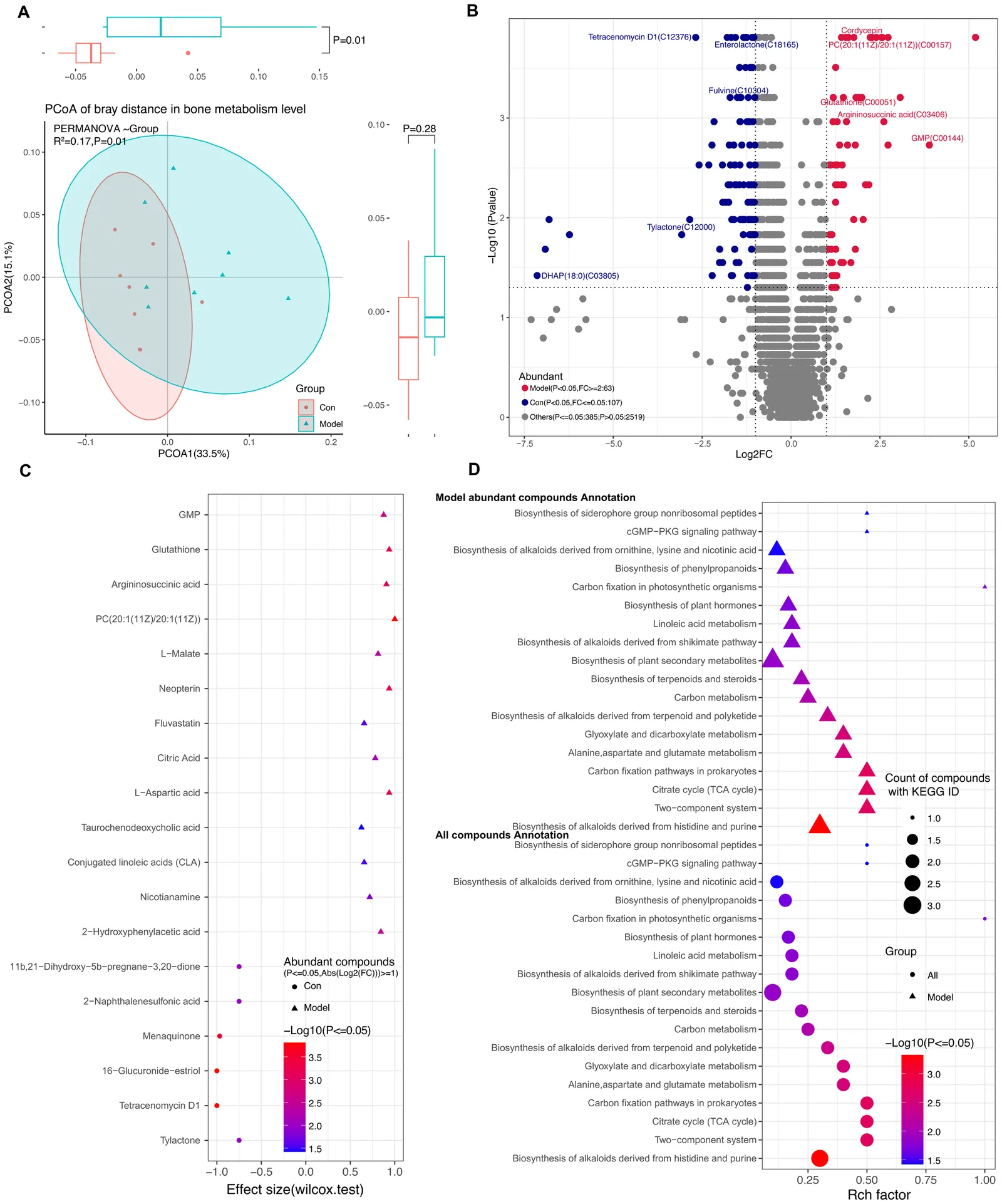
OVX-associated differences in the bone metabolome. **(A)** Bray–Curtis-based PCoA, with marginal boxplots showing sample-coordinate distributions. The overall group difference was significant by PERMANOVA (*R*² = 0.17, *P* = 0.01). **(B)** Volcano plot of differential metabolite features; 63 features were more abundant, and 107 were less abundant in OVX mice under the applied screening criteria. **(C)** Effect-size ranking of differential bone metabolite features. **(D)** KEGG annotation of all annotated metabolites and of metabolites more abundant in OVX; the horizontal axis denotes the rich factor. The cGMP–PKG entry is a KEGG pathway annotation and does not represent a direct measurement of pathway activity. Significance encodings should be read as −log10(*P* value). *n* = 8 mice per group. Con denotes Sham, and Model denotes OVX.

Bone metabolomic profiles differed between the OVX and Sham groups (PERMANOVA, *R*² = 0.17, *P* = 0.01). PCoA1 and PCoA2 accounted for 33.5% and 15.1% of the variance, respectively. PCoA1 scores differed between groups (*P* = 0.01), whereas PCoA2 scores did not (*P* = 0.28; **Figure 9A**). Using the fold-change (FC) thresholds FC ≥ 2 or FC ≤ 0.5 and *P* < 0.05, differential analysis identified 63 metabolites with higher relative abundance and 107 with lower relative abundance in OVX mice (**Figure 9B**). Among the representative metabolite features displayed in **Figure 9C**, guanosine monophosphate (GMP), glutathione, argininosuccinic acid, PC(20:1(11Z)/20:1(11Z)), L-malate, neopterin, fluvastatin, citric acid, L-aspartic acid, taurochenodeoxycholic acid, conjugated linoleic acids, nicotianamine, and 2-hydroxyphenylacetic acid were higher in OVX mice, whereas menaquinone, 16-glucuronide-estriol, tetracenomycin D1, and tylactone were higher in Sham mice. The OPLS-DA score plot showed separation between the OVX and Sham groups (**Supplementary Figure 16**). KEGG annotations of the metabolites higher in OVX mice and of the complete annotated metabolite set included the citrate cycle (TCA cycle), alanine, aspartate, and glutamate metabolism, glyoxylate and dicarboxylate metabolism, carbon metabolism, linoleic acid metabolism, biosynthesis of terpenoids and steroids, and the cGMP–PKG signaling pathway (**Figure 9D**). The measured GMP feature is distinct from cGMP and does not represent an increase in cGMP concentration or a direct measurement of cGMP–PKG pathway activity. For bone metabolites, Spearman correlations were examined across Sham and OVX mice. Body weight was positively correlated with L-aspartic acid (*ρ* = 0.715, *P* = 0.00186) and GMP (*ρ* = 0.709, *P* = 0.00211) relative abundances and inversely correlated with kynurenine (*ρ* = −0.626, *P* = 0.00941) and α-linolenic acid (*ρ* = −0.729, *P* = 0.00134) relative abundances. Positive correlations were observed between *Turicibacter* relative abundance and 16-glucuronide-estriol relative abundance (*ρ* = 0.788, *P* = 2.86 × 10⁻^4^) and between *Helicobacter rodentium* relative abundance and kynurenine relative abundance (*ρ* = 0.805, *P* = 1.71 × 10⁻^4^). An inverse correlation was observed between *Faecalibaculum* relative abundance and glutathione relative abundance (*ρ* = −0.797, *P* = 2.18 × 10⁻^4^). *Limosilactobacillus reuteri* relative abundance was positively correlated with N-acetyl-L-glutamic acid relative abundance (*ρ* = 0.688, *P* = 0.00320), whereas *Lactobacillus intestinalis* relative abundance was inversely correlated with glutathione relative abundance (*ρ* = −0.658, *P* = 0.00557; **Supplementary Figure 17**).

## 4 Discussion

This study compared OVX and Sham mice at a shared endpoint across systemic phenotypes and multiple omics layers. Transcriptomic profiling was performed in the colon, liver, and skeletal muscle; untargeted metabolomic profiling in colonic contents, liver, and bone; and targeted bile acid and short-chain fatty acid profiling in serum. The liver was examined at both the transcriptomic and metabolomic levels, whereas measurements from the gut, circulation, skeletal muscle, and bone provided complementary coverage across compartments. OVX-associated patterns varied among tissues and included correlations between taxa and metabolites, taxa and genes, and body weight and several molecular features. Together, these findings describe multitissue molecular differences associated with OVX and identify cross-omic relationships for further investigation.

Lower serum E2 concentrations in OVX mice occurred alongside inflammatory changes, skeletal remodeling, and increased body weight. Within the estrogen-dependent immune–bone axis (16), higher MCP-1 and TNF-α concentrations were consistent with the inflammatory response to estrogen loss (17), whereas lower PINP and OCN concentrations together with higher CTX-1 and TRAP-5b concentrations reflected a shift in bone turnover toward resorption (18). This marker profile was accompanied by deterioration in bone structure detected by micro-CT. Because bone-turnover responses vary across the postoperative course (19), these biochemical and structural findings place the present phenotype within the 9-week postoperative window. The sustained increase in body weight accords with OVX-associated weight gain and estrogen-sensitive hepatic metabolism reported in C57BL/6J mice (20, 21). Body weight was interpreted as part of the OVX-associated systemic phenotype, and the primary OVX–Sham comparisons represent overall group differences that include weight-related variation (22). This physiological background provides the context for interpreting the OVX-associated microbial and molecular patterns observed across the gut, circulation, liver, skeletal muscle, and bone.

Lower Shannon, Simpson, and inverse Simpson α-diversity and altered community composition in OVX mice are consistent with experimental and menopausal studies linking estrogen status to gut microbial ecology (9, 23). Among the taxa with lower relative abundances in OVX mice, colonization of gnotobiotic mice with individual *Turicibacter* strains produced strain-specific changes in bile-acid and host lipid profiles (24), *Lactobacillus johnsonii* P47-HY preserved barrier function in porcine intestinal epithelial cells challenged with enterotoxigenic *Escherichia coli* (25), and heat-killed *Limosilactobacillus reuteri* ATCC PTA 6475 increased BV/TV and trabecular thickness in OVX mice (26). Conversely, *Alistipes* and *Helicobacter ganmani* were enriched in OVX mice. *Alistipes* has been associated with metabolic and inflammatory phenotypes (27), whereas experimental *Helicobacter ganmani* colonization increased the cecal IL-12/23p40 mRNA expression in IL-10-deficient mice (28). These taxonomic changes occurred alongside broad overlap in UniRef gene-family profiles, consistent with cross-species mapping showing taxonomic divergence and partial functional overlap between mouse and human gut microbiomes (29). Spearman correlation analysis across Sham and OVX mice showed inverse correlations of body weight with the relative abundances of *Turicibacter*, *Lactobacillus johnsonii*, *Lactobacillus intestinalis*, and *Limosilactobacillus reuteri*. Body-weight findings from other models were strain dependent: *Turicibacter* sp. KKT8 reduced weight gain (30), non-viable *Lactobacillus johnsonii* JNU3402 reduced weight gain and the weights of several adipose depots (31), *Lactobacillus intestinalis* was inversely associated with weight gain and fat mass (32), and two *Limosilactobacillus reuteri* strains produced opposing effects on weight gain (33). Germ-free and recolonization experiments showed that sex steroid deficiency-associated trabecular bone loss was microbiota dependent (34). Microbiota transfer from OVX donors maintained on a low-fat diet increased recipient weight gain and altered hepatic expression of genes related to metabolism and inflammation (35), whereas fecal microbiota transplantation from healthy C57BL/6 mice increased intestinal ZO-1 and occludin expression, elevated fecal acetate and propionate concentrations, and attenuated bone loss in OVX mice (36). Studies using individual strains and whole microbial communities place the microbial differences observed here within the metabolic, intestinal, and skeletal responses to ovarian hormone loss.

OVX-associated differences in the colonic-content metabolome spanned tryptophan-related mucosal signaling, fermentation-derived metabolites, and bile-acid biology. Features annotated as histamine and 5-HIAA, a major serotonin catabolite, were lower in OVX mice. Microbially derived histamine can modulate mucosal immune responses (37), whereas 5-HIAA can enhance aryl hydrocarbon receptor (AhR)-responsive gene expression and epithelial barrier integrity and reduce inflammatory responses (38). In the combined-group analysis, *Turicibacter* relative abundance was positively correlated with the histamine-annotated feature; both were lower in OVX mice. Additional features annotated as L-tryptophan, serotonin, N-acetylserotonin, and 3-methylindole represented the serotonin and indole branches of tryptophan metabolism (39). Related studies provide a skeletal context: supplementation with the microbial indole metabolites indole-3-acetic acid and indole-3-propionic acid reduced bone loss in OVX mice (40), and tryptophan-related serum metabolic differences have been reported in postmenopausal women with reduced bone mineral density (41). Within the fermentation-related component, acetate, propionate, butyrate, and 2-methylbutyrate concentrations were lower in OVX mice, whereas the 2-methylvalerate concentration was higher. SCFAs support colonocyte energy metabolism (42) and colonic regulatory T-cell homeostasis (43), whereas propionate and butyrate prevented OVX-induced bone loss in mice (44). The bile-acid profile included lower T-α-MCA and UDCA-3S and higher HDCA and 6-ketoLCA. T-α-MCA acts as a farnesoid X receptor (FXR) antagonist in mice (45), and glycolithocholic acid increased circulating regulatory T cells that promoted osteogenic differentiation in coculture in an OVX rat study (46). This tryptophan-related, SCFA, and bile-acid profile situates OVX-associated microbial variation alongside luminal signals relevant to mucosal homeostasis and bone remodeling.

Colonic transcriptomic differences between OVX and Sham mice centered on epithelial organization, secretory-cell homeostasis, and metabolic and hormone-responsive processes, consistent with previous reports of intestinal responses to ovariectomy (4). Enrichment of epithelial-polarity and brush-border gene sets in Sham mice is relevant to the organization of the absorptive surface and directional nutrient transport (47). Enrichment of endoplasmic reticulum (ER) protein processing placed secretory-cell homeostasis within the same transcriptional response, consistent with the role of XBP1-dependent ER homeostasis in maintaining intestinal secretory cells and regulating inflammatory responses (48). Gene sets related to bile secretion, AMPK signaling, and estrogen signaling extended this response to nutrient handling, energy sensing, and sex-steroid responsiveness; sex-steroid deficiency has been linked to altered intestinal permeability and inflammation (34). Reduced expression of *Pck1*, *Acsl1*, and *Hsd3b3* further aligned the colonic response with glucose-, lipid-, and steroid-related processes. Higher *Dbp* expression added a circadian component (49), higher *Trpv6* expression was relevant to intestinal calcium transport (50), and higher *Grem1* expression to bone morphogenetic protein (BMP) antagonism during epithelial repair (51). Cross-layer correlation analysis further showed positive associations of *Turicibacter* relative abundance with *Pck1* expression and of *D. freteri* relative abundance with *Acsl1* expression. These relationships place microbial variation and colonic metabolic gene expression within a shared intestinal context. The combined gut microbial, colonic-content metabolomic, and colonic transcriptomic findings characterize an OVX-associated intestinal phenotype in which microbial variation, luminal metabolism, and host transcription co-occur, providing an intestinal context for the broader multiorgan molecular profile.

Serum concentrations of acetate, propionate, butyrate, and valerate were lower in OVX mice. Microbial fermentation is a major source of SCFAs, which contribute to host energy metabolism and immune regulation (42). In mouse studies, propionate and butyrate inhibited osteoclast differentiation and bone resorption (44), and butyrate promoted bone formation through regulatory T-cell-mediated WNT10B signaling (52). Of the 15 serum BAs that differed between groups, 14 were lower in OVX mice, whereas THDCA had a higher concentration. Circulating BA composition reflects hepatic synthesis and conjugation, intestinal reabsorption, and microbial transformation (53, 54). T-β-MCA acts as an intestinal FXR antagonist in mice and is influenced by microbial metabolism (45). Experimental studies have implicated FXR and bile acid receptor-1 (TGR5) signaling in bone remodeling (55), and total serum BA concentrations have been positively associated with bone mineral density and inversely associated with β-CTX in postmenopausal women (56). Across compartments, acetate, propionate, and butyrate concentrations were consistently lower in OVX mice. Human stable-isotope tracing has characterized the systemic availability of these SCFAs, and gut–liver exchange measurements have shown substantial hepatic uptake of butyrate (57, 58). The combined-group analysis identified a positive correlation between *Turicibacter* relative abundance and serum butyrate concentration, as well as an inverse correlation between *D. dubosii* relative abundance and serum T-β-MCA concentration. Both correlations followed the corresponding OVX–Sham group patterns, linking the circulating SCFA and BA profiles with gut microbial variation at the shared endpoint.

The hepatic metabolomic profile suggests that ovariectomy was accompanied by changes in both intermediary metabolism and BA composition. Features more abundant in OVX mice were annotated mainly to carbon, cofactor, and amino acid metabolism, whereas those more abundant in Sham mice were annotated mainly to fatty acid metabolism. These metabolic domains are influenced by hepatic estrogen receptor-α (ERα), which contributes to lipid and amino acid homeostasis after ovarian hormone loss (20). Prolonged estrogen deficiency has also been linked to impaired hepatic fatty acid oxidation and oxidative stress (59). The BA findings add a gut–liver component to this metabolic response. Most differential hepatic BAs had lower concentrations in OVX mice, whereas HDCA, TCA-3S, and GCA-3S concentrations were higher. Among the lower BAs, T-β-MCA is relevant to gut–liver signaling because it is an intestinal FXR antagonist whose abundance is influenced by microbial metabolism (45); both T-α-MCA and T-β-MCA derive from *Cyp2c70*-dependent muricholic acid synthesis in mice (60). The lower TUDCA is also relevant to hepatic stress biology, as pharmacological TUDCA reduces hepatic ER stress and injury in experimental steatohepatitis (61). The higher sulfated conjugates TCA-3S and GCA-3S add an elimination-related aspect to the BA profile because sulfation increases water solubility and facilitates elimination (62). These BA differences encompass compounds related to FXR signaling, muricholic acid synthesis, hepatic stress responses, and sulfation. Hepatic synthesis and conjugation, enterohepatic recycling, and microbial transformation collectively shape BA composition (53, 54). Within this framework, the NorCA concentration was lower in OVX mice across colonic contents, liver, and serum, whereas T-α-MCA concentration was lower and HDCA concentration was higher in both colonic contents and liver. Related taxon–metabolite correlations included positive associations between *Faecalibaculum* relative abundance and hepatic UCA content and between *A. caecimuris* relative abundance and hepatic cholestenone relative abundance; in both pairs, the taxon and hepatic metabolite were lower in OVX mice. The cross-compartment BA directions and taxon–metabolite relationships were consistent with an associative enterohepatic pattern at the shared endpoint.

Hepatic gene-set findings indicated an estrogen-sensitive transcriptional response involving lipoprotein transport, nutrient metabolism, and protein regulation. In OVX mice, enrichment of gene sets related to high-density lipoprotein (HDL) particles, chylomicrons, ATPase-dependent transport, ABC transporters, and fatty acid, carbohydrate, and amino acid metabolism, including branched-chain amino acid degradation, emphasized transport and nutrient metabolism. This pattern is relevant to estrogen-dependent hepatic lipid handling because hepatocyte estrogen receptor-α supports HDL-mediated reverse cholesterol transport in female mice fed a Western-type diet, and E2 treatment beginning at ovariectomy reduced hepatic lipid deposition during high-fat feeding (63, 64). In Sham mice, enrichment of gene sets related to deubiquitination, ubiquitin ligase-related processes, palmitoylation, and ribosomal, lysosomal, and intracellular transport functions emphasized protein modification, turnover, and trafficking. The metabolic relevance of these processes is illustrated by USP14-mediated deubiquitination, which stabilizes FASN, and CD36 palmitoylation, which regulates its cellular distribution and fatty acid uptake in hepatocytes (65, 66). The OVX- and Sham-enriched profiles therefore represent different regulatory layers that converge on hepatic lipid handling, with nutrient transport and metabolism prominent in OVX mice and protein stability, localization, and turnover prominent in Sham mice. At the taxon–gene level, *L. intestinalis* relative abundance was positively correlated with hepatic *Cyp3a41a* expression. In mice, hepatic *Cyp3a41a* expression is female-predominant and transcriptionally activated by HNF4α (67), providing a sex-dependent hepatic context for this relationship. The pathway contrast indicates that ovarian hormone status is associated with hepatic regulation at metabolic and protein-processing levels, whereas the *L. intestinalis*–*Cyp3a41a* relationship associates gut microbial variation with female-predominant hepatic gene expression.

The skeletal-muscle response to OVX was organized around three biological themes: matrix–receptor organization, inflammatory and hormone-responsive signaling, and muscle metabolic homeostasis. The prominence of extracellular matrix–receptor interaction and receptor- and membrane-related terms is biologically relevant because the myofiber–matrix interface supports force transmission, cell adhesion, and tissue repair (68). Cytokine binding, inflammatory mediator regulation of transient receptor potential channels, NOD-like receptor signaling, and estrogen signaling brought inflammatory and ovarian-hormone-responsive processes into the same transcriptional pattern. This interpretation is supported by studies showing E2/ERα-dependent regulation of skeletal-muscle transcription in OVX mice (69) and ovarian-hormone-dependent changes in phosphorylation networks governing metabolism, calcium signaling, and sarcomeric organization (70). Insulin signaling, translation, autophagy, muscle cytoskeleton, and circadian regulation further linked the transcriptomic response to muscle maintenance and metabolic control. Basal autophagy maintains muscle mass and myofiber integrity (71), whereas the intrinsic muscle clock regulates insulin sensitivity and glucose metabolism (72). Among the taxon–gene findings, inverse correlations were observed between *D. freteri* relative abundance and skeletal-muscle *Plcb4* expression and between *Turicibacter* relative abundance and *Prkag3* expression. *Prkag3* encodes the muscle-enriched AMPKγ3 subunit, a regulator of glucose uptake and glycogen replenishment during recovery from exercise (73). Together, the skeletal-muscle transcriptomic and taxon–gene findings extend the multitissue OVX pattern to matrix organization, inflammatory signaling, and muscle energy metabolism.

Bone metabolomic profiles differed between OVX and Sham mice, adding molecular context to the skeletal phenotype. Similar bone-tissue metabolic remodeling has been reported in an independent OVX study (74). Within a broader annotation pattern spanning the TCA cycle, amino-acid metabolism, and lipid metabolism—metabolic themes also identified in human osteoporosis studies (75)—features annotated as citric acid and L-malate were more abundant in OVX bone. Citrate contributes to cellular energy metabolism and bone mineralization. Experimental loss of *Slc13a5* function reduced citrate uptake and disrupted mineral-nodule formation in osteoblasts, whereas osteoblast-specific deletion in mice compromised cortical thickness and bone strength (76). Redox- and nucleotide-related signals included higher glutathione- and GMP-annotated features. GMP and cGMP are distinct nucleotide species, and the separately annotated cGMP–PKG pathway has an established role in skeletal remodeling (77). Experimental studies have also linked glutathione-related redox processes to osteoblast differentiation and osteoclastogenesis (78, 79). The inverse association between *Faecalibaculum* relative abundance and the glutathione-annotated feature extended this redox theme to the gut microbiome. Among hormone- and bile-acid-related signals, the feature annotated as taurochenodeoxycholic acid was more abundant in OVX bone, whereas that annotated as 16-glucuronide-estriol was less abundant. More broadly, total serum bile-acid levels were positively associated with bone mineral density and inversely associated with β-CTX in postmenopausal women (56), whereas glycolithocholic acid administration reduced bone loss in OVX rats (46). Estriol 16-O-glucuronidation is a documented route of estrogen conjugation in humans (80). Within the same steroid-conjugate theme, *Turicibacter* relative abundance was positively associated with the 16-glucuronide-estriol–annotated feature. Together, these findings characterize OVX-associated skeletal remodeling across bone structure and turnover, local metabolic chemistry, and gut taxon–metabolite associations.

**Figure 10:**
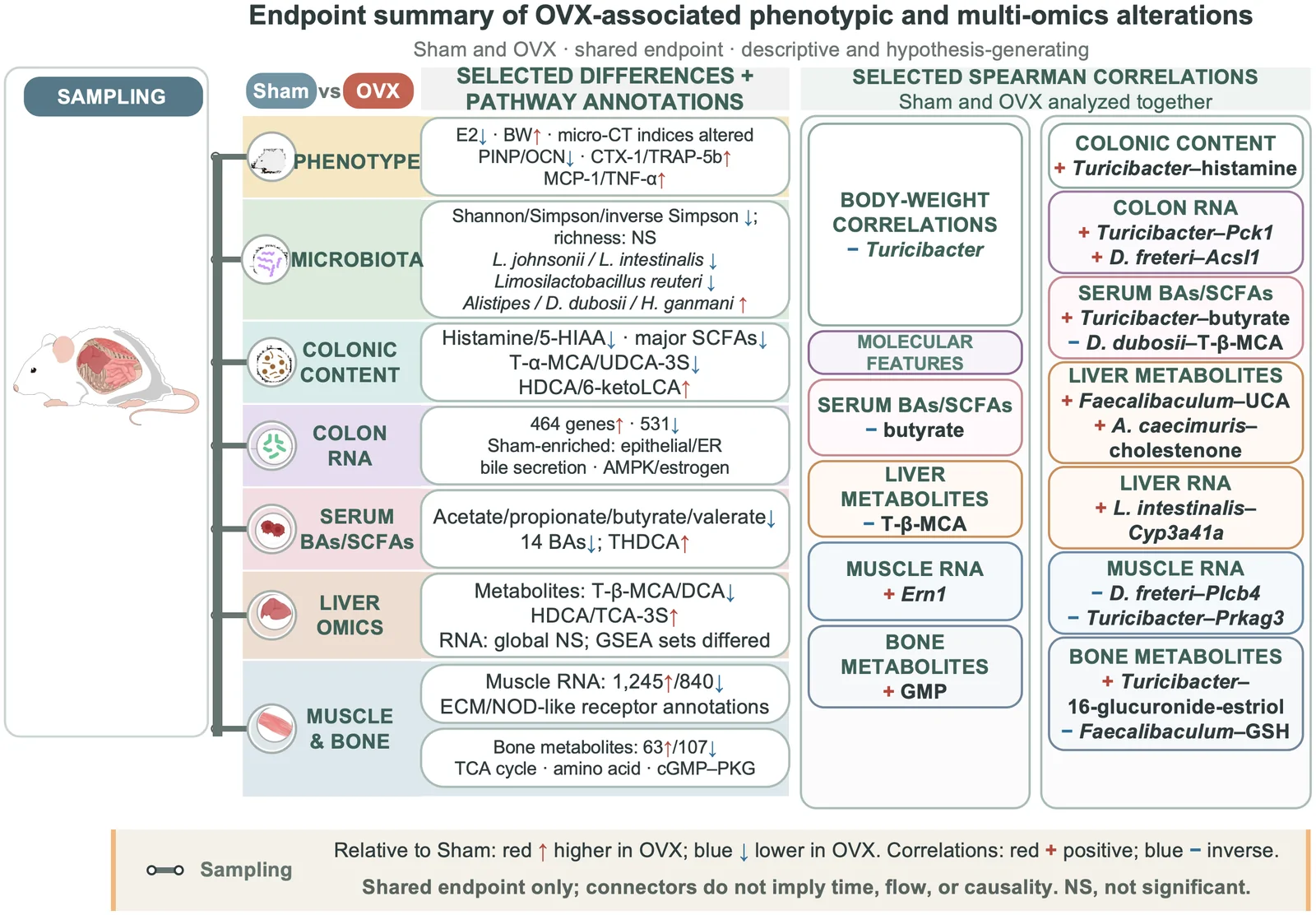
Integrative endpoint summary of OVX-associated multitissue findings. The schematic summarizes measured Sham–OVX differences, pathway annotations, and associations at the shared endpoint. Pathway labels, including ECM/NOD and cGMP–PKG, denote enrichment annotations rather than direct measurements of pathway activity. Upward and downward arrows in the group-comparison section indicate higher and lower values, concentrations, relative abundances, or expression in OVX mice, as appropriate. Associations in the correlation sections were evaluated using Spearman correlation analysis; red plus signs indicate positive associations, and blue minus signs indicate inverse associations. The schematic is organized by data layer; connectors summarize common sampling or association structure and do not indicate temporal sequence or inter-organ flow. These associations do not imply causality.

**Figure 10** integrates the OVX–Sham phenotypic differences and molecular patterns across the gut, circulation, liver, skeletal muscle, and bone at the shared endpoint with body-weight-centered and taxon–omics correlations across the measured data layers. When Sham and OVX mice were analyzed together, body weight was inversely correlated with *Turicibacter* relative abundance and serum butyrate concentration. These correlation directions paralleled the group differences, as OVX mice had higher body weight but lower *Turicibacter* relative abundance and serum butyrate concentration. The taxon–omics component summarizes microbial–metabolite and microbial–gene correlations across compartments.

This study has several limitations. Because omics and tissue samples were collected at a single endpoint, the findings describe concurrent differences. Assessment of temporal ordering and causal mediation requires longitudinal or intervention studies. Given the sample size relative to data dimensionality, cross-layer analyses focused on pairwise correlations, whereas higher-order multivariable and network analyses would benefit from larger datasets. Body composition, terminal tissue weights, and bone mineral density were outside the scope of the present measurements. Shotgun metagenomics reflects microbial functional potential rather than active transcription. Bulk RNA-seq provides tissue-level expression profiles, and single-cell or other cell-resolved approaches are suited to examining cell-type-specific responses. Surgical OVX in young mouse models abrupt ovarian hormone loss, whereas natural menopause involves a gradual endocrine and age-related transition. Species differences in gut microbial ecology and bile acid metabolism also shape the interpretation of individual taxa and metabolites. Future studies can combine orthogonal confirmation of metabolite annotations with targeted molecular validation and direct microbiota or metabolite interventions to evaluate the functional relevance of these findings.

## 5 Conclusion

Integrative multiomics analysis of the OVX model characterized endocrine, inflammatory, and skeletal phenotypes alongside differences in gut microbial composition and molecular profiles across multiple tissues. Several SCFA differences were directionally concordant between colonic contents and serum, whereas BA differences spanned colonic contents, serum, and liver. Molecular patterns varied by tissue and data layer, encompassing transcriptional differences in colon and skeletal muscle and metabolomic variation in liver and bone. In the liver, ranked-list GSEA further highlighted gene-set patterns related to lipid transport, nutrient metabolism, and protein processing. Spearman correlation analysis across Sham and OVX mice identified associations between gut taxa and metabolites or tissue gene expression, as well as between body weight and microbial or molecular features. These findings delineate concurrent OVX-associated differences across the gut, circulation, liver, skeletal muscle, and bone and generate cross-layer hypotheses for testing temporal ordering, cellular context, and functional relevance.

## Data Availability

The datasets presented in this study can be found in online repositories. The names of the repository/repositories and accession number(s) can be found below: https://db.cngb.org/search/project/CNP0004326/, CNP0004326.

## Supplementary material

Supplementary Figure 1 Quantitative femoral micro-CT analysis in Sham and OVX mice. Boxplots show bone volume fraction (BV/TV), trabecular thickness (Tb.Th), trabecular number (Tb.N), trabecular separation (Tb.Sp), and Cs.Th. *n* = 6 mice per group. *P* values are from two-sided Wilcoxon rank-sum tests and are shown above the brackets (Tb.Th, *P* = 0.015; all other parameters, *P* = 0.0022). Model denotes OVX.

Supplementary Figure 2 Additional ordination analyses of metagenomic profiles. **(A)** Genus-level Bray–Curtis PCoA; the overall group difference was evaluated by PERMANOVA (*R*² = 0.133, *P* = 0.045). **(B)** Genus-level non-metric multidimensional scaling (NMDS; stress = 0.041). **(C)** Species-level NMDS (stress = 0.045). **(D)** UniRef functional-profile PCoA; the global group comparison was not significant by PERMANOVA (*R*² = 0.037, *P* = 0.48). Marginal boxplots show the distributions of sample coordinates on the displayed axes. *n* = 8 mice per group. Con denotes Sham and Model denotes OVX.

Supplementary Figure 3 Relative-abundance composition of the gut microbiota in colonic contents. Stacked bars show **(A)** phylum-level composition, **(B)** the 30 most abundant genera, and **(C)** the 30 most abundant species in the Sham and OVX groups. Asterisks mark taxa with *P* < 0.05 in the displayed between-group comparisons. *n* = 8 mice per group. Con denotes Sham and Model denotes OVX.

Supplementary Figure 4 LEfSe linear discriminant analysis (LDA) score ranking of taxa that discriminated the Sham and OVX groups. Taxa are ordered by LDA score; point shape indicates the group in which each taxon was enriched, and color encodes −log10(*P* value). *n* = 8 mice per group. Con denotes Sham and Model denotes OVX. LEfSe used *P* < 0.05 and an LDA score threshold of 2.0.

Supplementary Figure 5 Pairwise correlation matrix for differential gut microbial taxa and body weight. Spearman correlation analysis was performed across Sham and OVX mice (*n* = 16). Colors represent correlation coefficients, with red indicating positive and purple indicating inverse correlations. Symbols denote the displayed two-sided *P*-value thresholds (**P* < 0.05; ^+^*P* < 0.01). The displayed correlation *P* values were not adjusted for multiple comparisons.

Supplementary Figure 6 Supervised orthogonal partial least-squares discriminant analysis (OPLS-DA) score plot of colonic-content metabolomic profiles in Sham and OVX mice. Each point represents one mouse, and the dashed ellipses summarize the group distributions in score space. *n* = 8 mice per group. Con denotes Sham and Model denotes OVX.

Supplementary Figure 7 Effect-size ranking of differential colonic-content metabolite features. Features are ranked by the Wilcoxon effect size; point color encodes −log10(*P* value), point size encodes the OPLS-DA variable importance in projection (VIP) score, and point shape indicates the group with the higher relative abundance. *n* = 8 mice per group. Con denotes Sham and Model denotes OVX.

Supplementary Figure 8 Correlation heatmap between differential gut microbial taxa and differential colonic-content metabolite features. The column labeled “Weight” represents body weight (g). Spearman correlation analysis was performed across Sham and OVX mice (*n* = 16). Colors represent correlation coefficients, with red indicating positive and purple indicating inverse correlations. Symbols denote the displayed two-sided *P*-value thresholds (**P* < 0.05; +*P* < 0.01). The displayed correlation *P* values were not adjusted for multiple comparisons.

Supplementary Figure 9 Ranked-list GSEA of Gene Ontology (GO) terms in the colonic transcriptome. Enrichment curves show the displayed Sham-enriched GO terms; the inset table reports nominal and adjusted *P* values. *n* = 13 mice (7 Sham and 6 OVX).

Supplementary Figure 10 Ranked-list GSEA of KEGG pathways in the colonic transcriptome. Enrichment curves show the displayed Sham-enriched KEGG pathways; the inset table reports nominal and adjusted *P* values. *n* = 13 mice (7 Sham and 6 OVX).

Supplementary Figure 11 Correlation heatmap between differential gut microbial taxa and displayed colonic pathway-related genes. The column labeled “Weight” represents body weight (g). Spearman correlation analysis was performed across Sham and OVX mice (*n* = 13). Colors represent correlation coefficients, with red indicating positive and purple indicating inverse correlations. Symbols denote the displayed two-sided *P*-value thresholds (**P* < 0.05; ^+^*P* < 0.01). The displayed correlation *P* values were not adjusted for multiple comparisons.

Supplementary Figure 12 Correlation heatmap between differential gut microbial taxa and serum SCFA and BA concentrations. The column labeled “Weight” represents body weight (g). Spearman correlation analysis was performed across Sham and OVX mice (*n* = 16). Colors represent correlation coefficients, with red indicating positive and purple indicating inverse correlations. Symbols denote the displayed two-sided *P*-value thresholds (**P* < 0.05; ^+^*P* < 0.01). The displayed correlation *P* values were not adjusted for multiple comparisons.

Supplementary Figure 13 Correlation heatmap between differential gut microbial taxa and hepatic metabolite features. The column labeled “Weight” represents body weight (g). Spearman correlation analysis was performed across Sham and OVX mice (*n* = 16). Colors represent correlation coefficients, with red indicating positive and purple indicating inverse correlations. Symbols denote the displayed two-sided *P*-value thresholds (**P* < 0.05; ^+^*P* < 0.01). The displayed correlation *P* values were not adjusted for multiple comparisons.

Supplementary Figure 14 Correlation heatmap between differential gut microbial taxa and displayed hepatic pathway-related genes. The column labeled “Weight” represents body weight (g). Spearman correlation analysis was performed across Sham and OVX mice (*n* = 13). Colors represent correlation coefficients, with red indicating positive and purple indicating inverse correlations. Symbols denote the displayed two-sided *P*-value thresholds (**P* < 0.05; ^+^*P* < 0.01). The displayed correlation *P* values were not adjusted for multiple comparisons.

Supplementary Figure 15 Correlation heatmap between differential gut microbial taxa and displayed skeletal-muscle pathway-related genes. The column labeled “Weight” represents body weight (g). Spearman correlation analysis was performed across Sham and OVX mice (*n* = 16). Colors represent correlation coefficients, with red indicating positive and purple indicating inverse correlations. Symbols denote the displayed two-sided *P*-value thresholds (**P* < 0.05; ^+^*P* < 0.01). The displayed correlation *P* values were not adjusted for multiple comparisons.

Supplementary Figure 16 Supervised OPLS-DA score plot of bone metabolomic profiles in Sham and OVX mice. Each point represents one mouse, and the dashed ellipses summarize the group distributions in score space. *n* = 8 mice per group. Con denotes Sham and Model denotes OVX.

Supplementary Figure 17 Correlation heatmap between differential gut microbial taxa and bone metabolite features. The column labeled “Weight” represents body weight (g). Spearman correlation analysis was performed across Sham and OVX mice (*n* = 16). Colors represent correlation coefficients, with red indicating positive and purple indicating inverse correlations. Symbols denote the displayed two-sided *P*-value thresholds (**P* < 0.05; ^+^*P* < 0.01). The displayed correlation *P* values were not adjusted for multiple comparisons.
