# Supplementary material for: Multiorgan and gut microbial alterations in ovariectomized mice: a multiomics analysis
Source: Front Immunol. 2026 Sep 9;17:1847257. doi: 10.3389/fimmu.2026.1847257 (PMC13597264; doi:10.3389/fimmu.2026.1847257)
Supplement: Supplementary file 1 [file Image1.pdf]

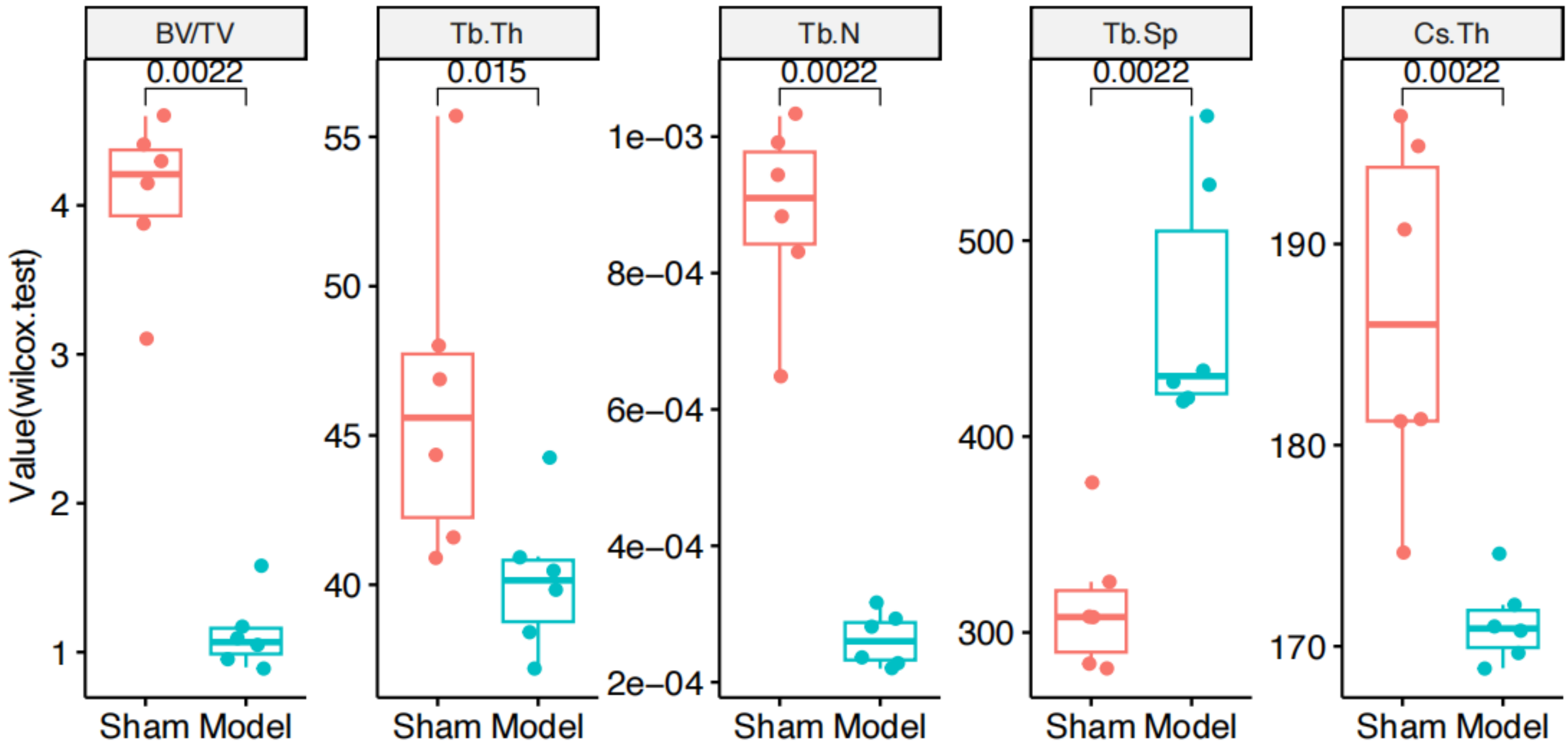

Supplementary_Figure_01_MicroCT_Quantification

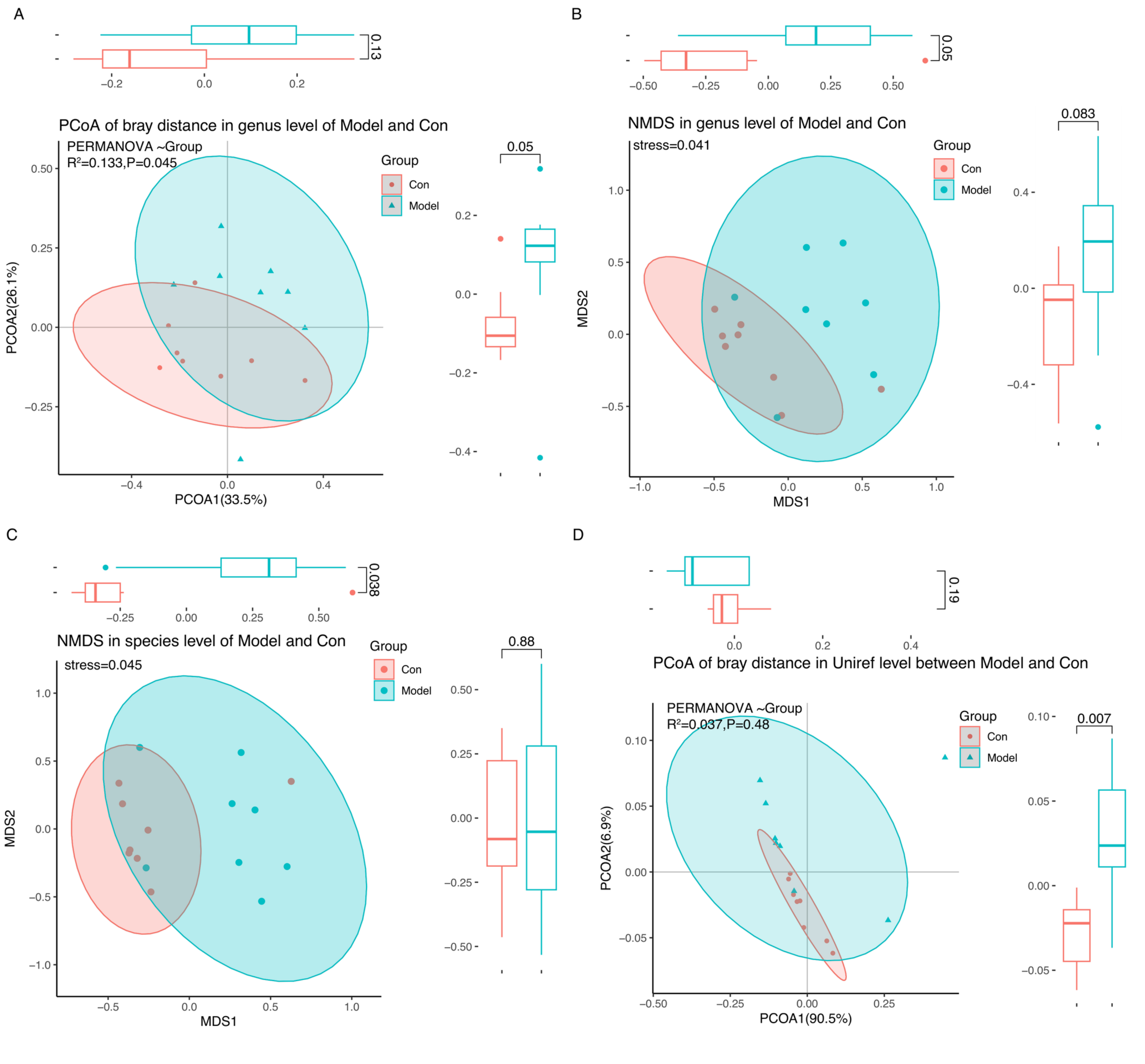

Supplementary_Figure_02_Metagenomic_Ordination

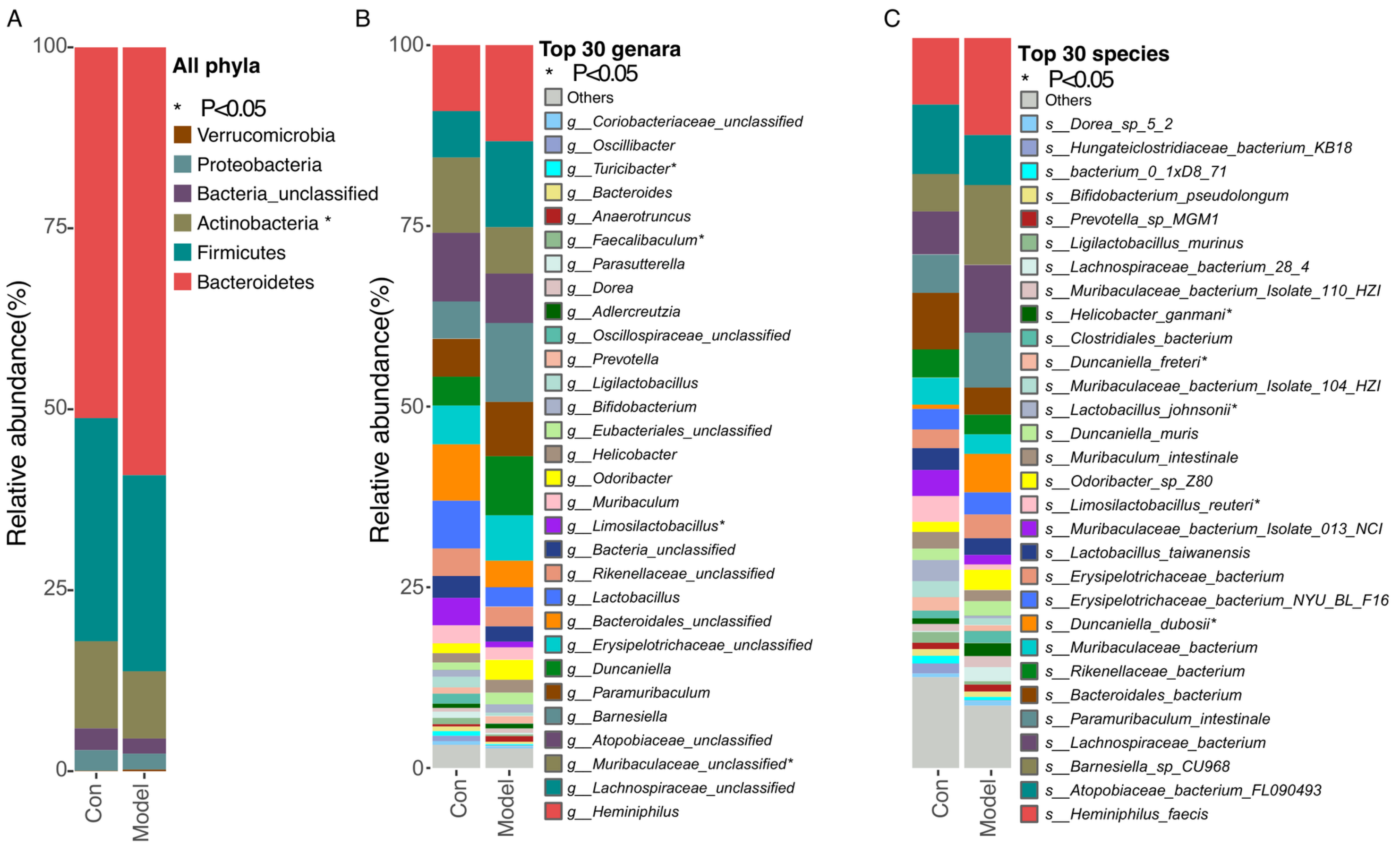

Supplementary_Figure_03_Gut_Taxonomic_Composition

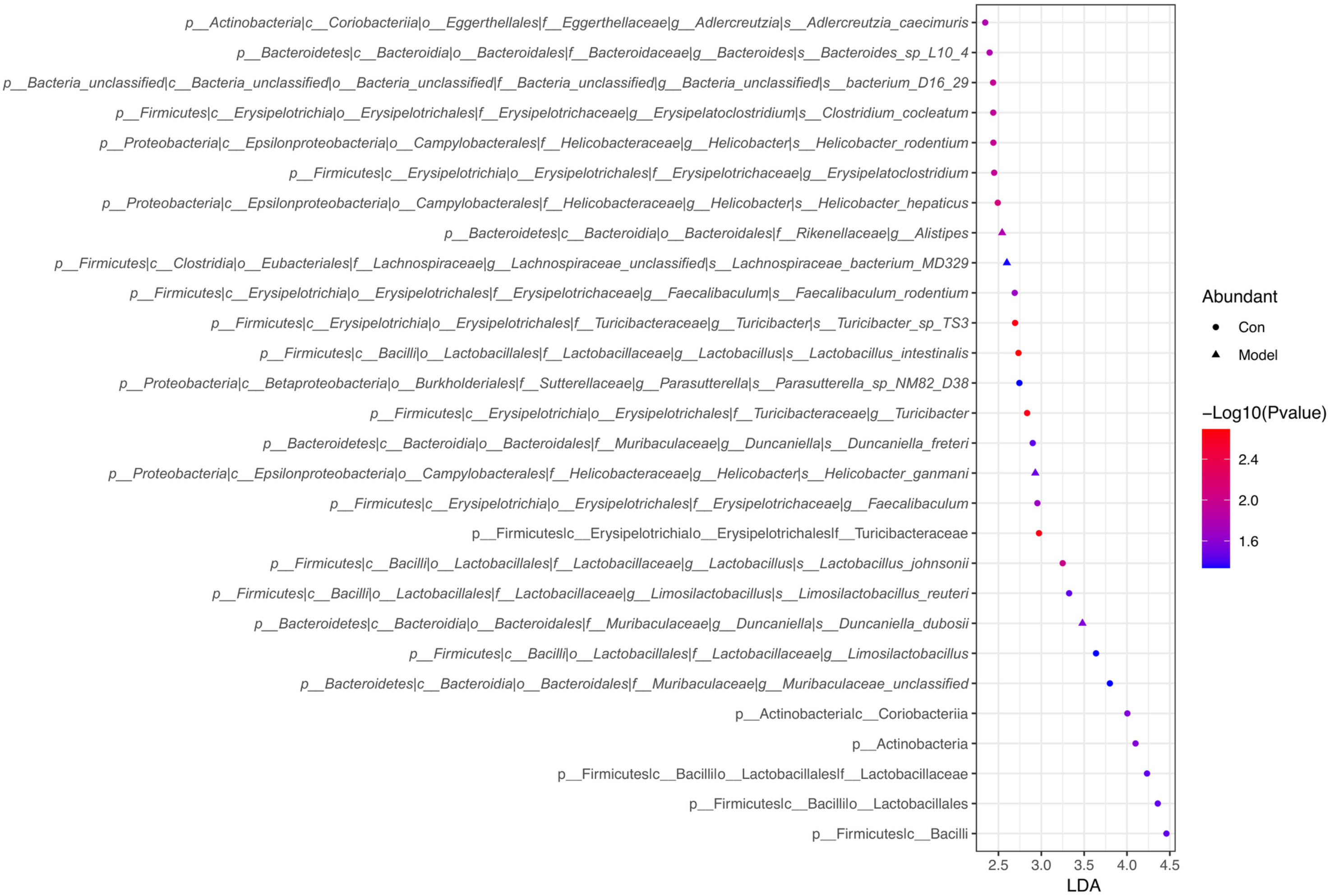

Supplementary_Figure_04_LEfSe_LDA_Differential_Taxa

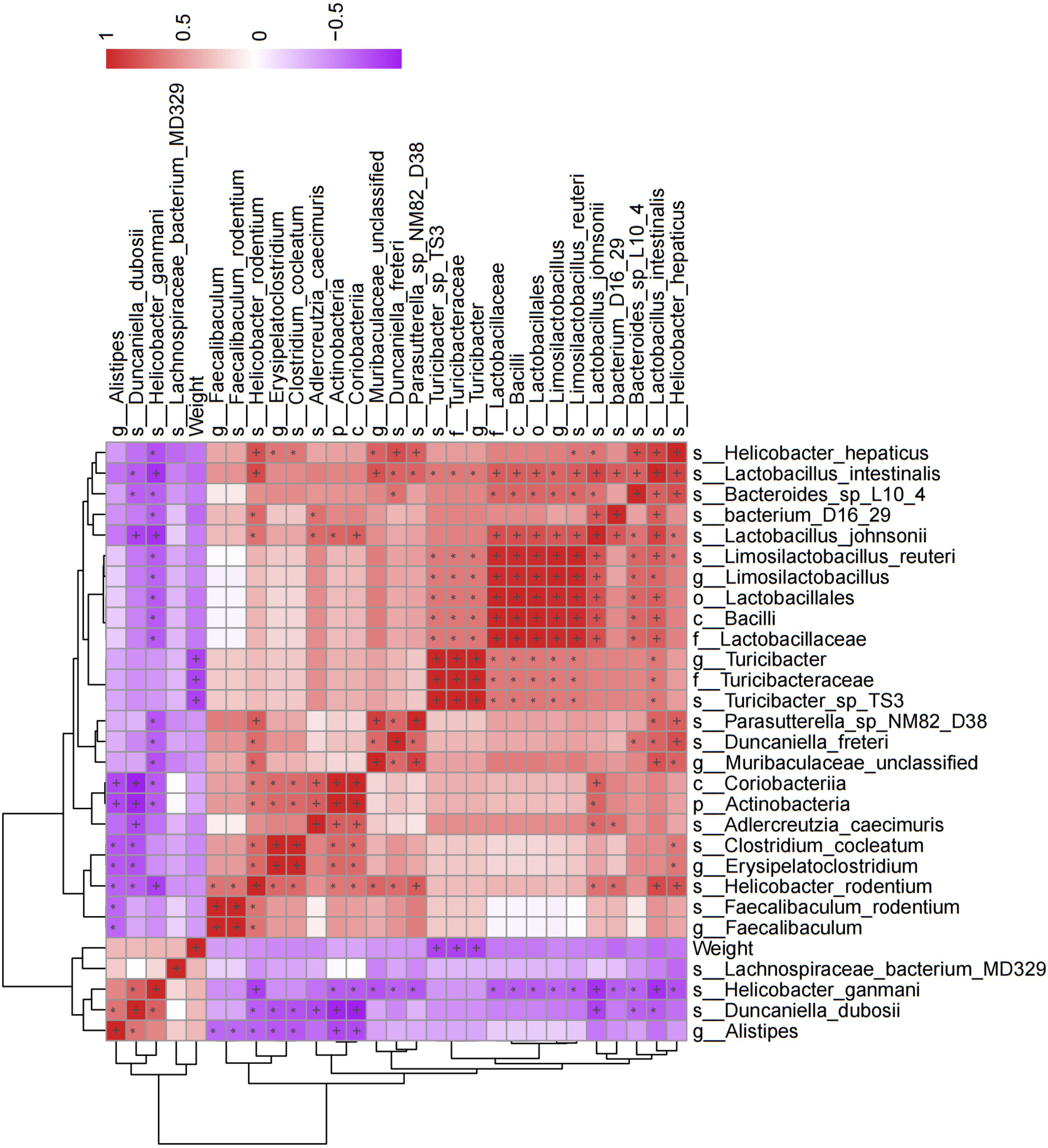

Supplementary_Figure_05_Taxa_Body_Weight_Correlation_Matrix_

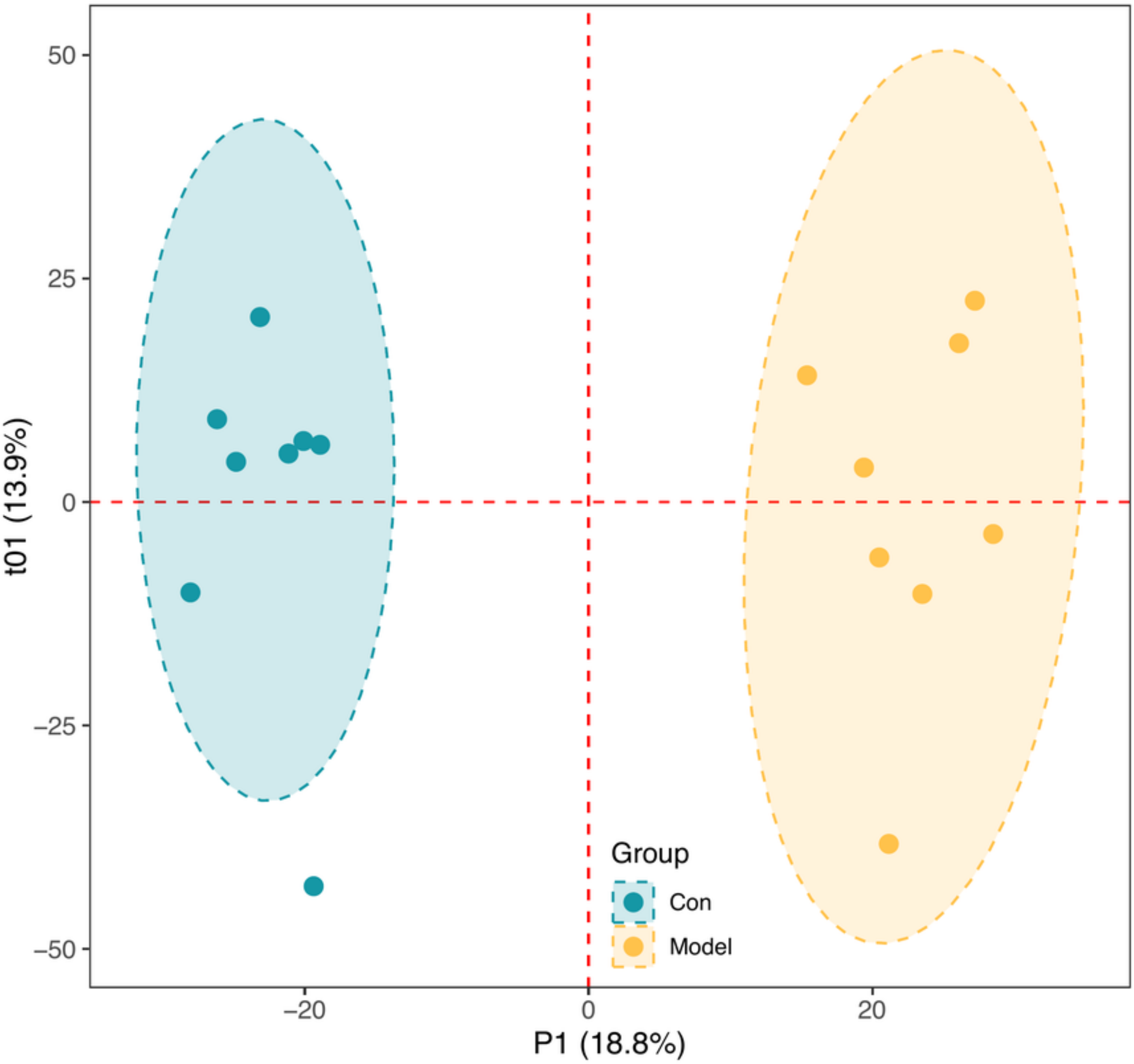

Supplementary_Figure_06_Colonic_Content_Metabolome_OPLS_DA

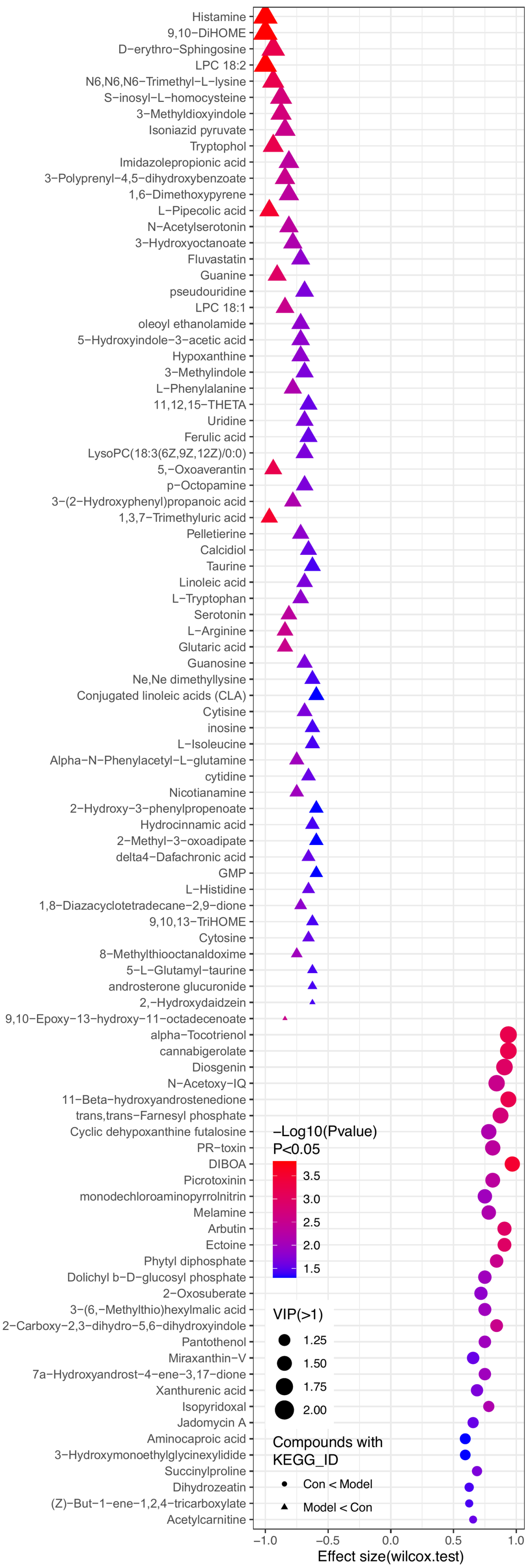

Supplementary_Figure_07_Colonic_Content_Metabolite_Feature_Ranking

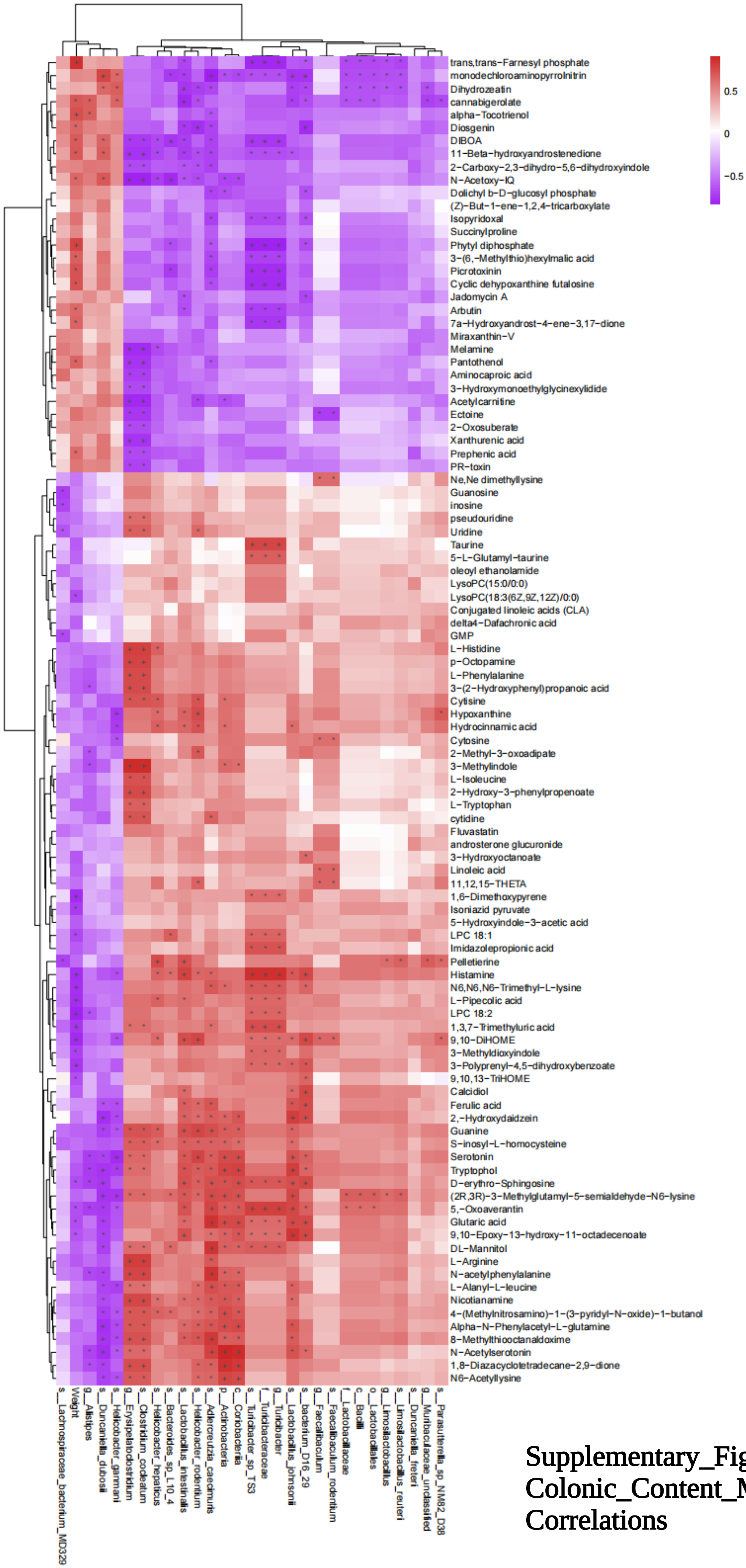

Supplementary_Figure_08_Taxa_Colonic_Content_Metabolite_Correlations

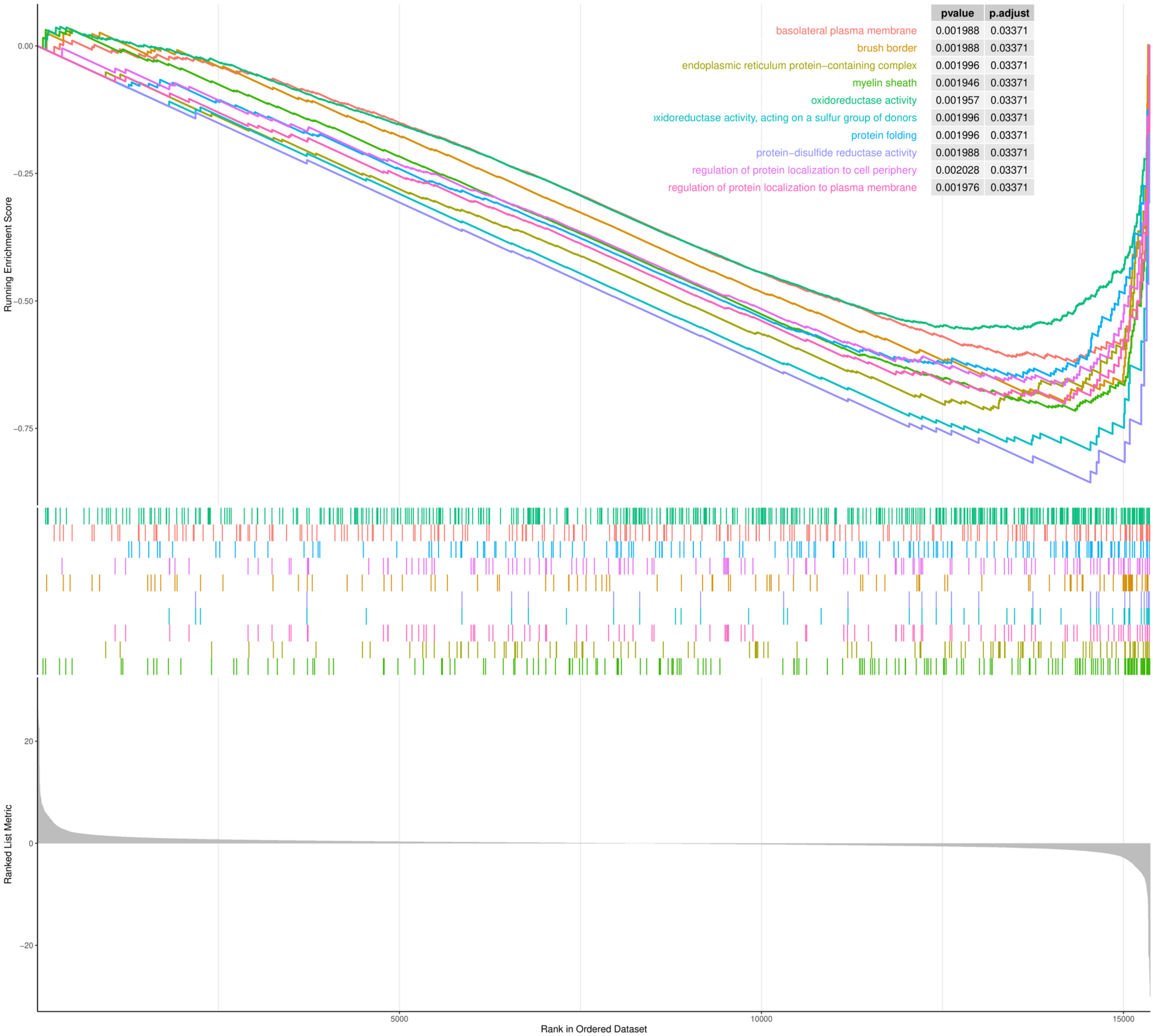

Supplementary_Figure_09_Colon_Transcriptome_GO_GSEA

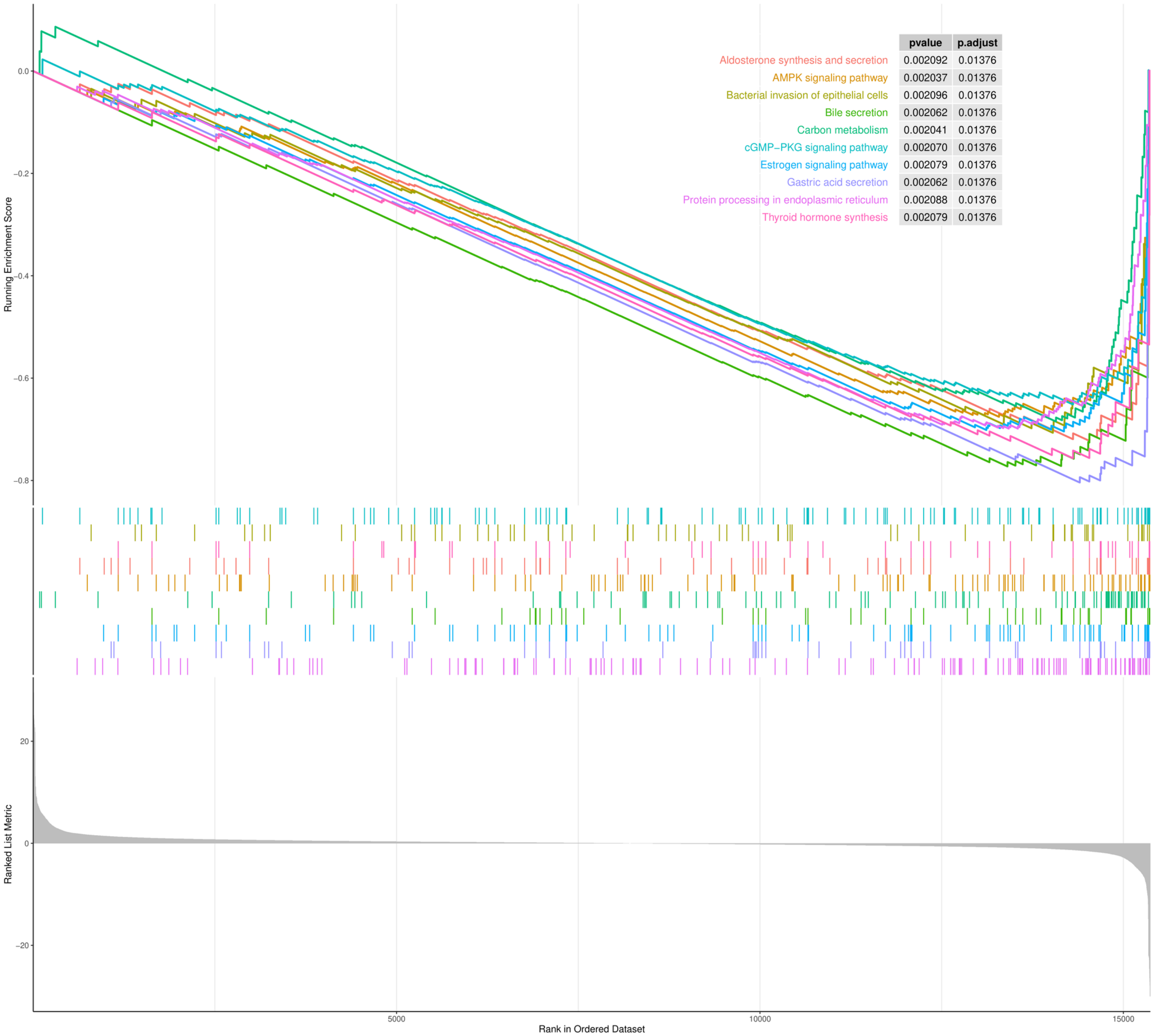

Supplementary_Figure_10_Colon_Transcriptome_KEGG_GSEA

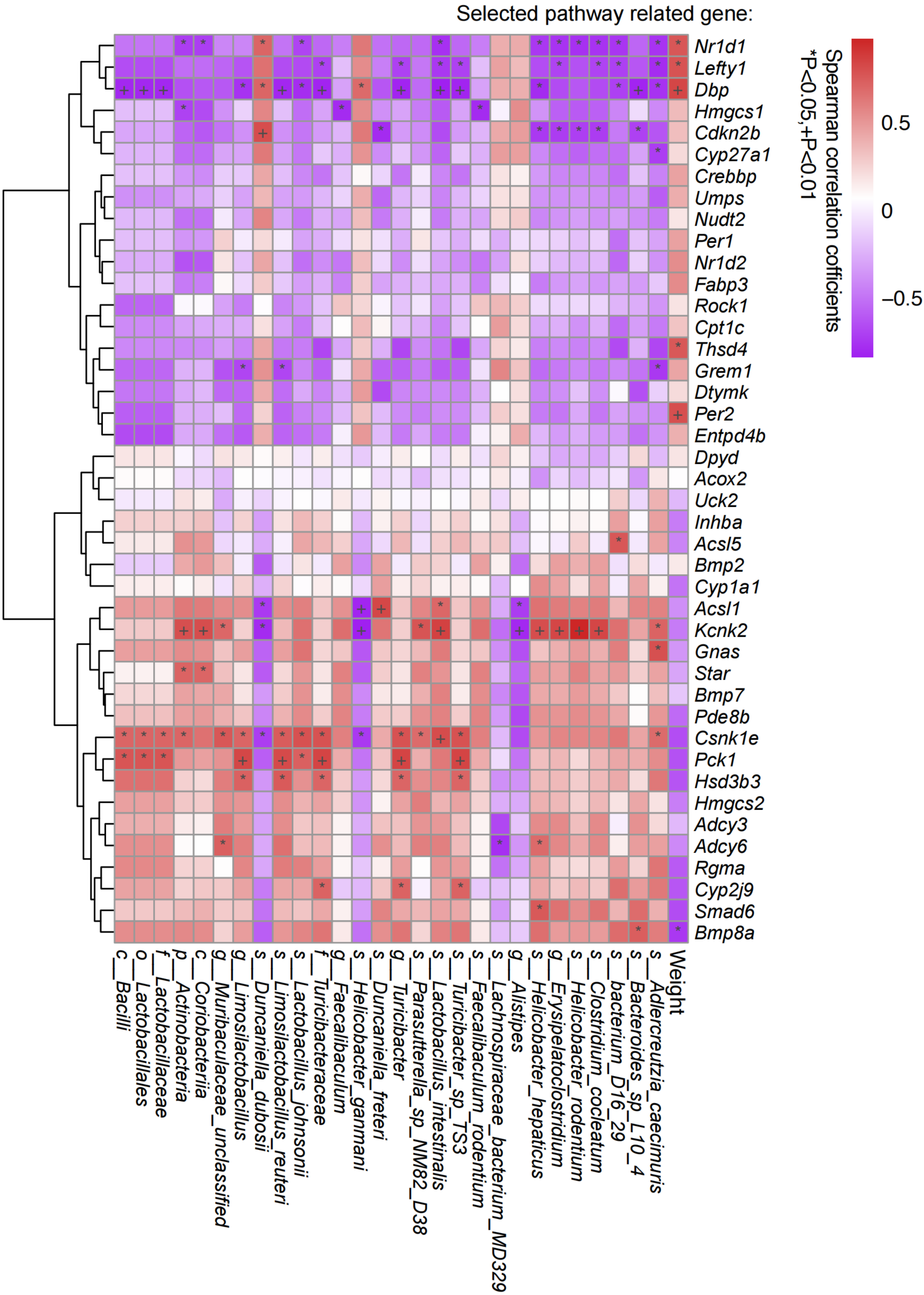

Supplementary_Figure_11_Taxa_Colon_Gene_Correlations

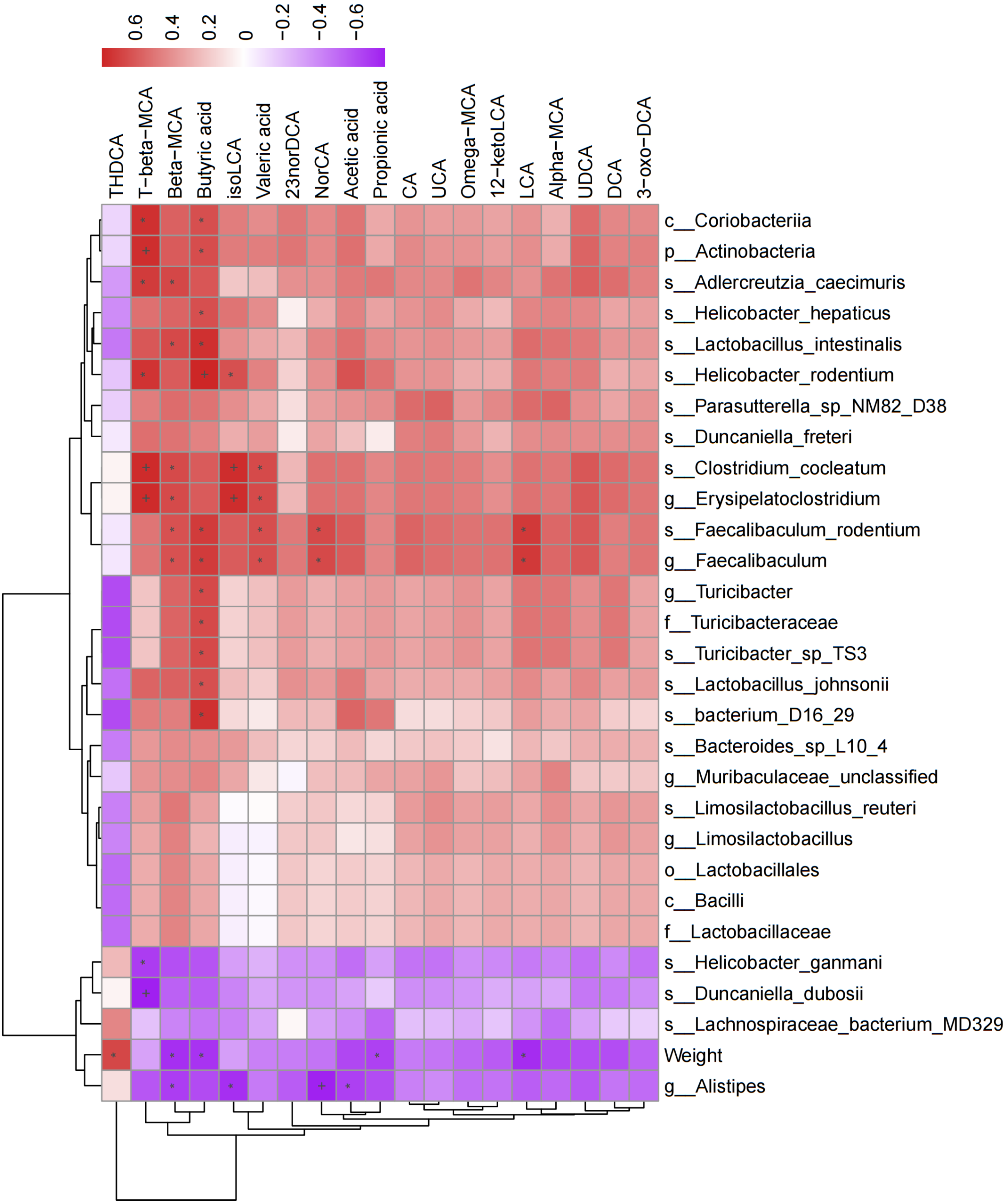

Supplementary_Figure_12_Taxa_Serum_SCFA_Bile_Acid_Correlations

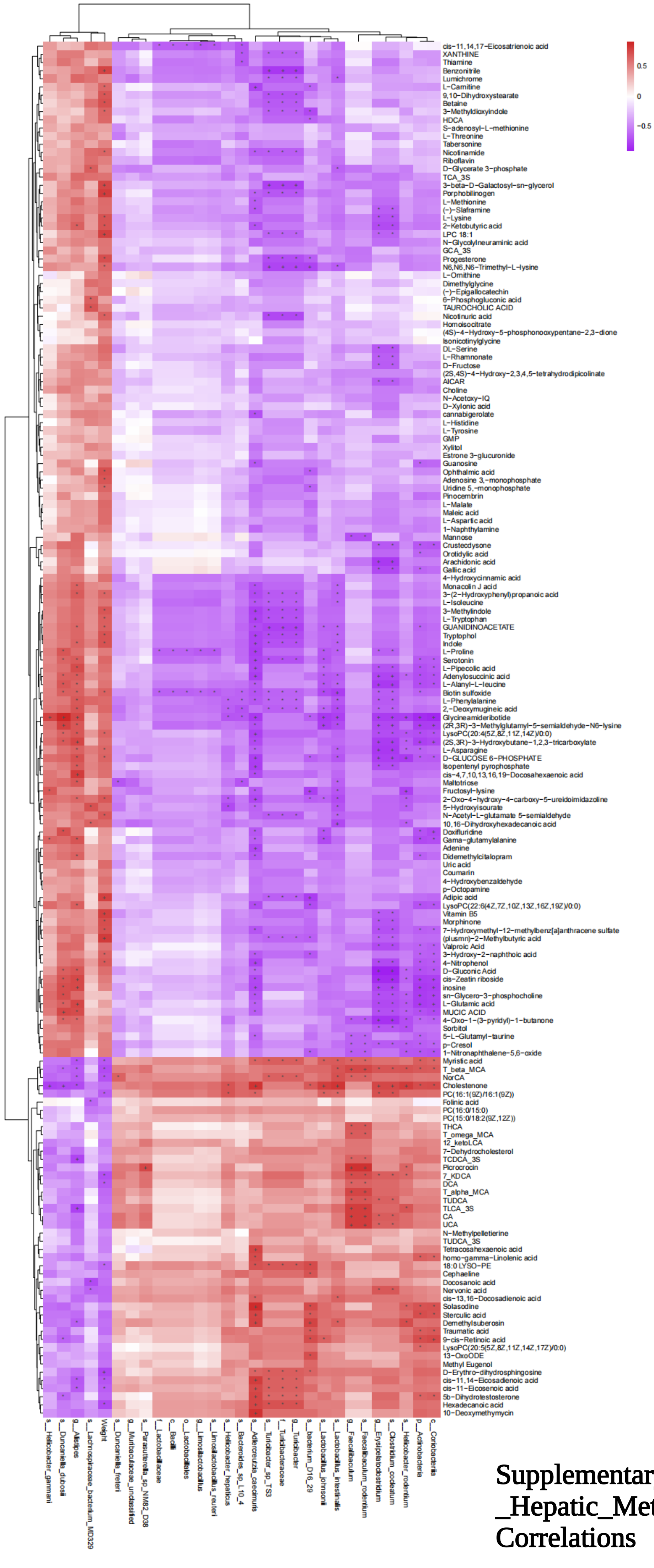

Supplementary_Figure_13_Taxa_Hepatic_Metabolite_Correlations

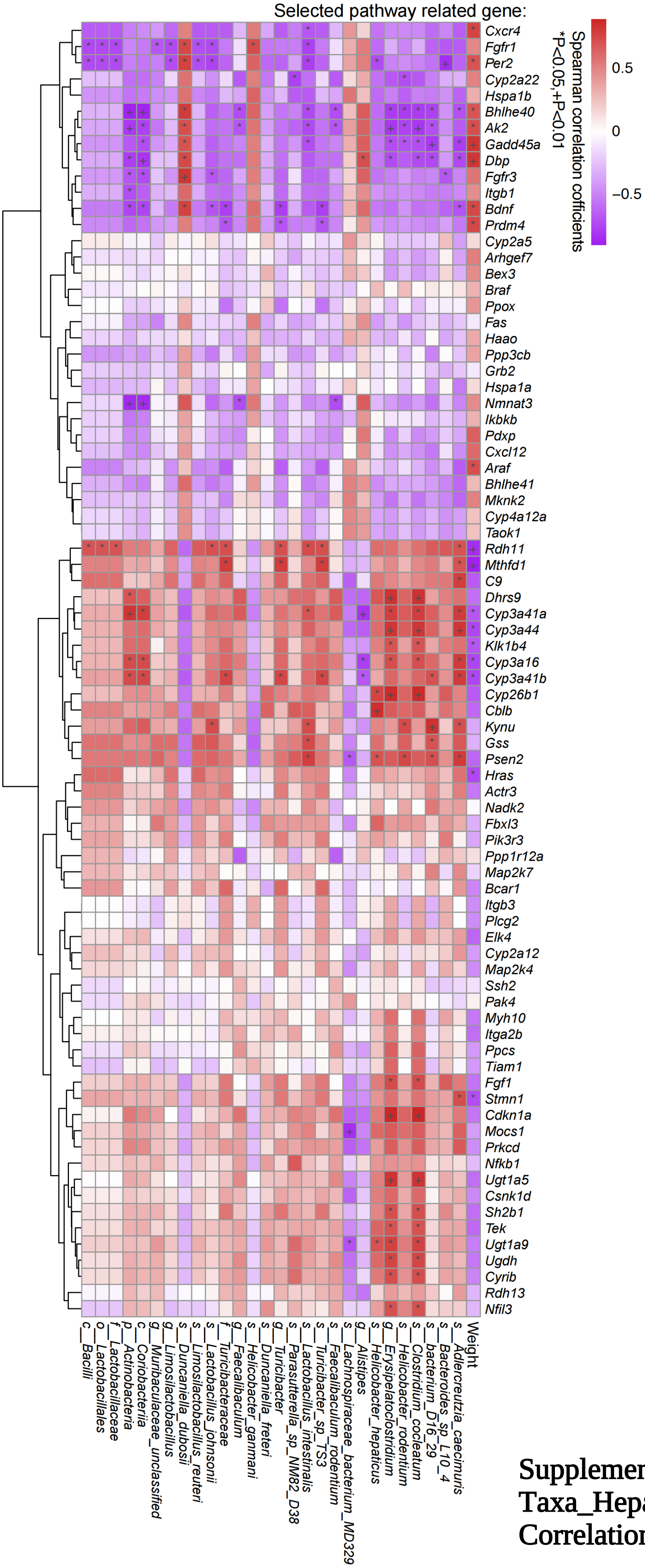

Supplementary_Figure_14_Taxa_Hepatic_Gene_Correlations

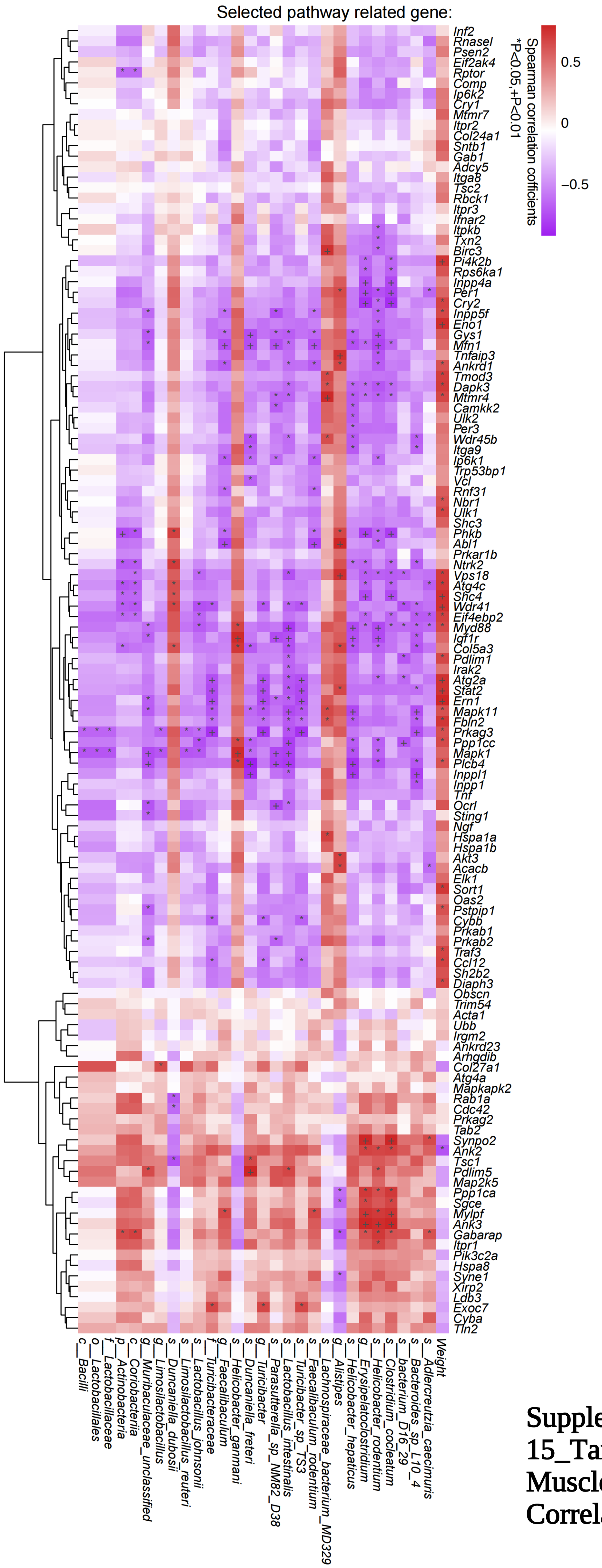

Supplementary_Figure_15_Taxa_Skeletal_Muscle_Gene_Correlations

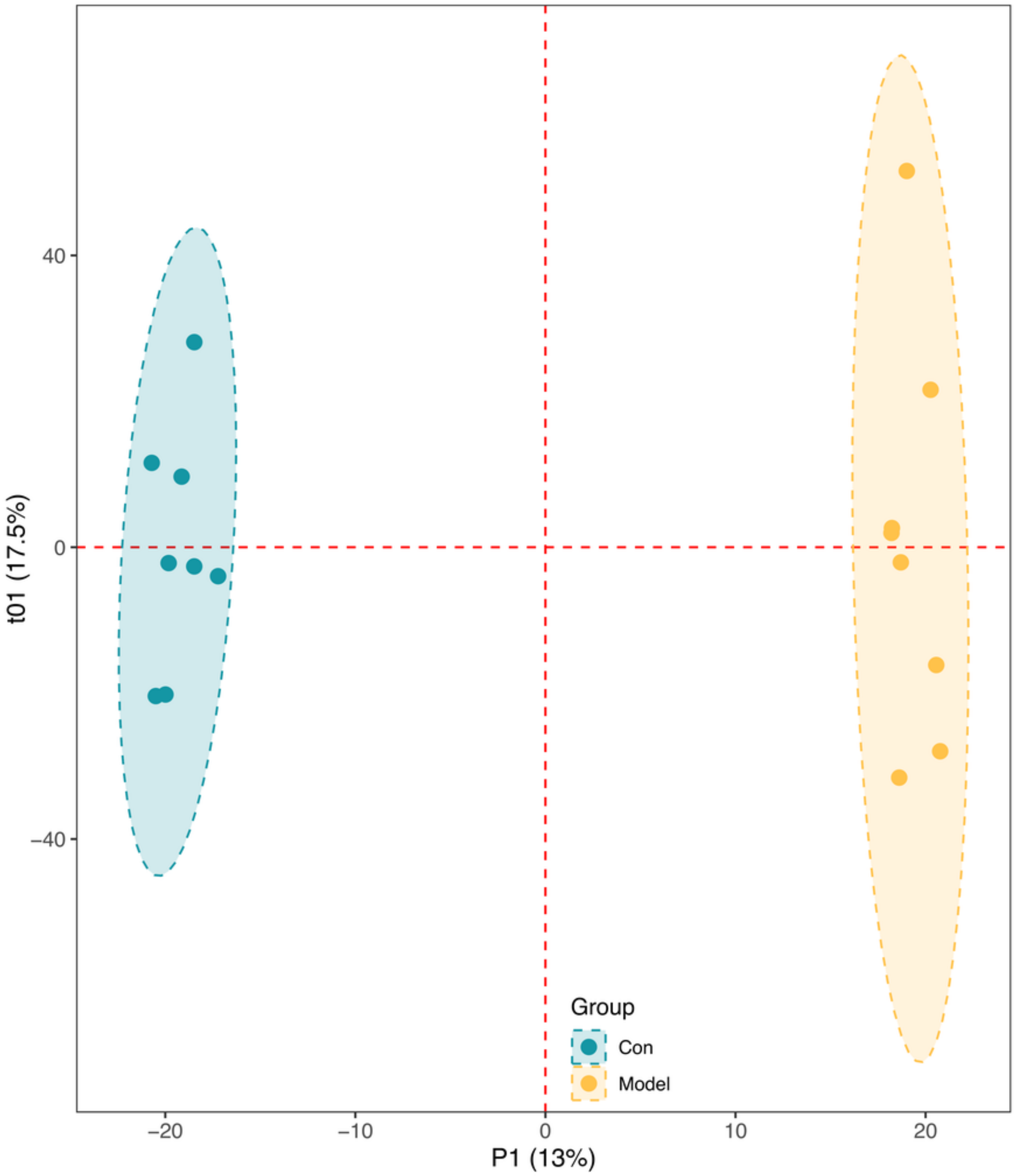

Supplementary_Figure_16_Bone_Metabolome_OPLS_DA

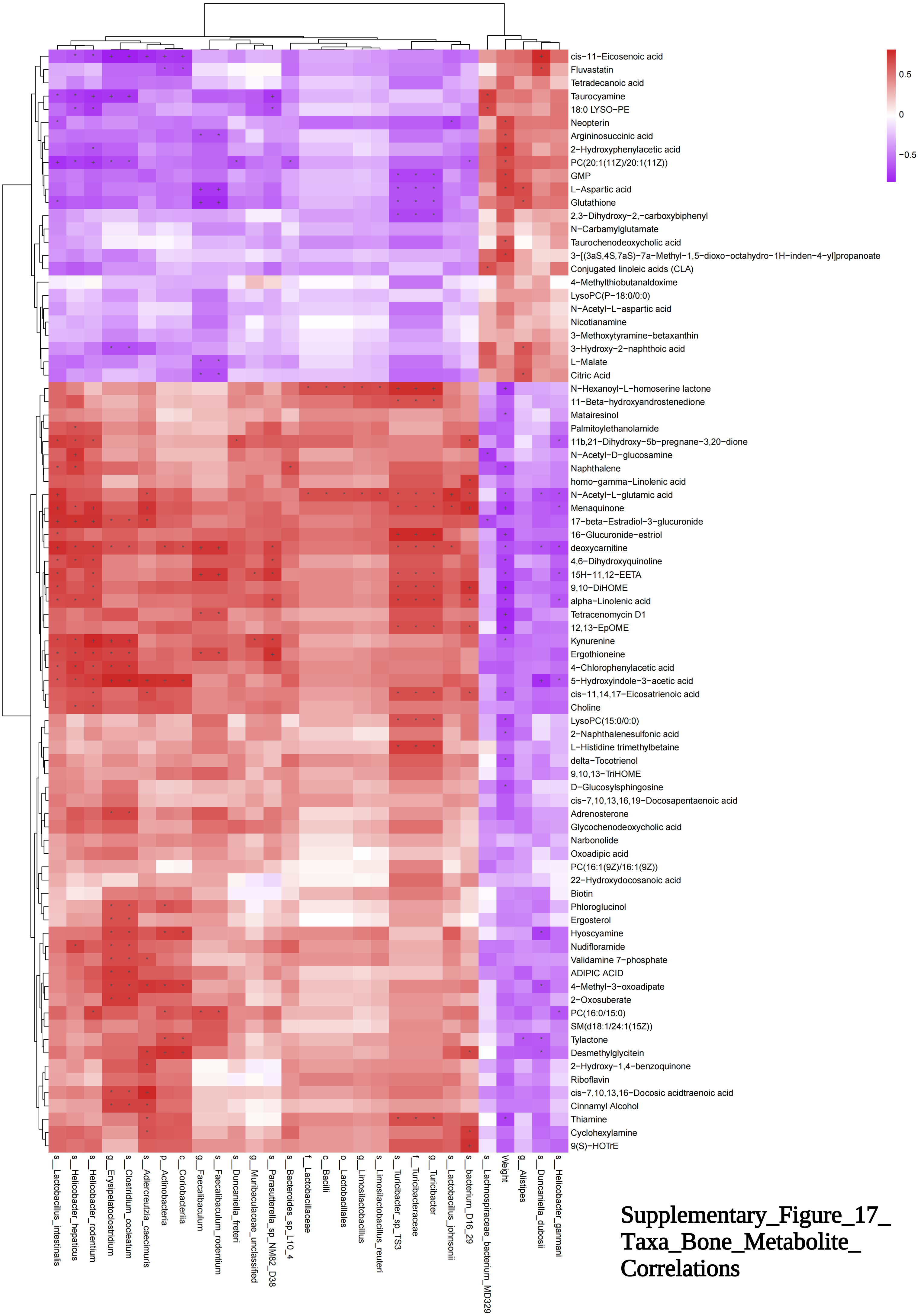

Supplementary_Figure_17_Taxa_Bone_Metabolite_Correlations
